# Albumin Precoating of Nanoparticles: A Reliable Stealth Strategy?

**DOI:** 10.1021/acsomega.6c04297

**Published:** 2026-08-17

**Authors:** Vinicius S. Guido, André F. Lima, Natasha Mina, Jackelinne Y. Hayashi, Ismael F. Lima, Leo K. Iwai, Maria Luiza V. Oliva, Fernando L. A. Fonseca, Alexandre K. Tashima, Alioscka A. Sousa

**Affiliations:** † Department of Biochemistry, 28105 Federal University of São Paulo (UNIFESP), São Paulo, São Paulo 04044-020, Brazil; ‡ Laboratory of Applied Toxinology, Center of Toxins, Immune-Response and Cell Signaling LETA/CeTICS, Butantan Institute, São Paulo, São Paulo 05503-900, Brazil; § Pharmaceutical Sciences Department, Federal University of São Paulo (UNIFESP), Diadema, São Paulo 09913-030, Brazil; ∥ Clinical Laboratory Department, Faculty of Medicine of ABC (FMABC), Santo Andre, São Paulo 09060-650, Brazil; ⊥ Laboratory of Mass Spectrometry Applied to Biomolecules, Federal University of São Paulo (UNIFESP), São Paulo, São Paulo 04039-032, Brazil

## Abstract

Albumin precoating has emerged as a strategy to impart nanoparticles (NPs) with stealth-like properties and as an alternative to NP surface PEGylation. Yet how such coatings influence protein corona formation and hemocompatibility in human blood remains poorly understood. To address this, we precoated model hydrophilic and hydrophobic polystyrene NPs with either fatted or defatted human serum albumin (HSA) and exposed them to human plasma and blood-based models. Proteomic analysis of the adsorbed protein corona revealed formulation-dependent differences in composition, but an overall favorable profile characterized by enrichment of dysopsonins (HSA and apolipoproteins) and depletion of major opsonins (complement proteins and immunoglobulins). Consistent with these findings, NP uptake by phagocytic blood cells was low across all formulations. However, evaluation in physiologically relevant human-derived models uncovered formulation-dependent biological responses. Specifically, three of the four HSA-precoated NPs exhibited complement activation or prothrombotic tendencies, whereas fatted HSA on hydrophobic NPs showed consistent blood compatibility across multiple assays. Taken together, these findings demonstrate that the hemocompatibility of albumin-coated NPs is determined by the interplay between NP core surface chemistry and albumin formulation, rather than by albumin precoating alone, providing important design criteria for albumin-based NP coatings.

## Graphical abstract

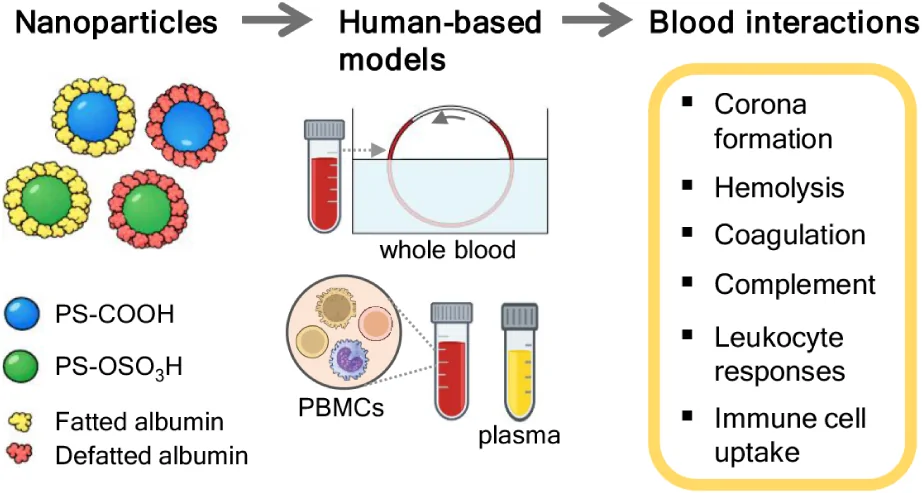

## Introduction

Nanoparticles (NPs) are widely explored for clinical applications, yet controlling their behavior in vivo remains a significant challenge due to the complexity of bio–nano interactions. Upon exposure to blood, NPs readily acquire a layer of adsorbed proteins and other biomolecules, collectively known as the biomolecular corona.^1,2^ The composition and structure of this corona give the NPs a biological identity and influence their interactions with blood components, often leading to immune recognition and rapid clearance by phagocytes.^3–7^ In more severe cases, corona formation can trigger acute thrombotic and inflammatory responses.^8–11^ Therefore, to minimize immune cell recognition and mitigate nanotoxicity risks, NPs can be rationally designed to modulate biomolecular corona formation. A common approach involves tailoring their surface chemistries with specialized coatings,^12–16^ most notably polyethylene glycol (PEG) and zwitterionic polymers.^12–14^ However, growing concerns over the immunogenicity and complement-activating potential of these polymeric materials highlight the need for safer and more reliable alternatives.^17–20^

As an alternative to surface PEGylation and related strategies, protein-based protective coatings have emerged as a promising approach.^21–26^ Among these, albumin precoating has shown particular potential. This strategy has been applied to liposomal, polymeric, and inorganic NPs, primarily to reduce nonspecific NP–protein interactions, limit immune cell recognition, and prolong circulation time in vivo.^27–36^ The advantages of albumin stem from its inherent biocompatibility, including noncytotoxicity, nonimmunogenicity, and biodegradability, along with its low cost, ready availability, and ease of use.^37,38^

However, it is crucial to recognize that albumin-coated NPs form a distinct nanostructure that differs fundamentally from free albumin; that is, the favorable attributes of albumin do not automatically carry over to the NPs. For example, albumin association with the NP surface may induce structural changes in the protein.^39–41^ Adding to this complexity, commercially available albumin exists in two major forms: fatty acid-bound (“fatted”) and fatty acid-free (“defatted”). Bound fatty acids are known to enhance the structural stability and rigidity of fatted albumin compared to its defatted counterpart.^42,43^ In addition, fatty acid binding modulates the electrostatic properties of albumin, including a slight reduction in its isoelectric point.^44,45^ Together, these physicochemical differences between fatted and defatted albumin could influence the bioresponses of NPs coated with these distinct protein variants. Another confounding factor is that most studies evaluating albumin-coated NPs have been conducted using animal cell lines and murine models. However, it is now widely recognized that the use of human-based models is essential for generating more realistic and biologically relevant data.^46–48^ For example, recent studies have demonstrated that NPs may exhibit stealth-like behavior in animal models yet still trigger innate immune activation when evaluated in human-derived systems.^20,47^

Therefore, despite the promise of albumin precoating strategies, it remains unclear how albumin-coated NPs interact with human blood components and how these interactions govern NP blood compatibility. To address this gap, here we systematically investigate the blood interactions of albumin-coated NPs, using hydrophilic and hydrophobic polystyrene NPs as model systems, in combination with fatted and defatted human serum albumin (HSA) variants. We initially assess how NP surface properties and HSA type influence the physicochemical characteristics of the resulting HSA-precoated NPs. Then, we apply detailed proteomic analysis to identify the unique plasma protein signatures adsorbed onto each preformed HSA coating. Blood compatibility is investigated under physiologically relevant conditions using an ex vivo human whole-blood model, which preserves the natural interplay among blood cascade systems and cells. These whole-blood assays are further supplemented by nanotoxicity studies in isolated plasma and blood cells. Together, these investigations provide a comprehensive assessment of how albumin precoating shapes protein corona formation and NP blood compatibility.

## Results and Discussion

### Preparation and Characterization of HSA-Precoated NPs

We precoated polystyrene NPs with either fatted or defatted HSA (HSA-f and HSA-df, respectively). As model systems, we employed 100 nm polystyrene NPs with two distinct surface chemistries: hydrophilic carboxyl-modified (PS-COOH) and hydrophobic sulfate-modified (PS-OSO_3_H), both of which are commonly used in nano–bio interaction studies.^49–51^ The resulting four HSA-precoated NP formulations are illustrated in Figure 1A. Hereafter, we refer to bare PS-COOH as C-Uncoated, while the corresponding coated NPs are termed C-HSA-f and C-HSA-df. Likewise, bare PS-OSO_3_H is designated S-Uncoated, and the coated forms are S-HSA-f and S-HSA-df. Surface coating was achieved through passive adsorption by incubating the NPs with HSA for 24 h. The resulting coated NPs were characterized for size and zeta potential (Figure 1B–C). We observed an increase in hydrodynamic diameter and a reduction in the magnitude of negative zeta potential values, suggesting the formation of stable coatings in all cases. We also measured the amount of adsorbed albumin through the bicinchoninic acid assay for protein quantification, finding nearly 500 molecules per NP (Figure 1D). Considering uncertainties in NP concentration and minor losses during centrifugation, this value is reasonably consistent with the theoretical estimate of 800 molecules required to form a protein monolayer. Collectively, these results demonstrate that the precoated NPs exhibited comparable size, surface charge, and coating density, irrespective of NP core type or HSA variant.

**Figure 1:**
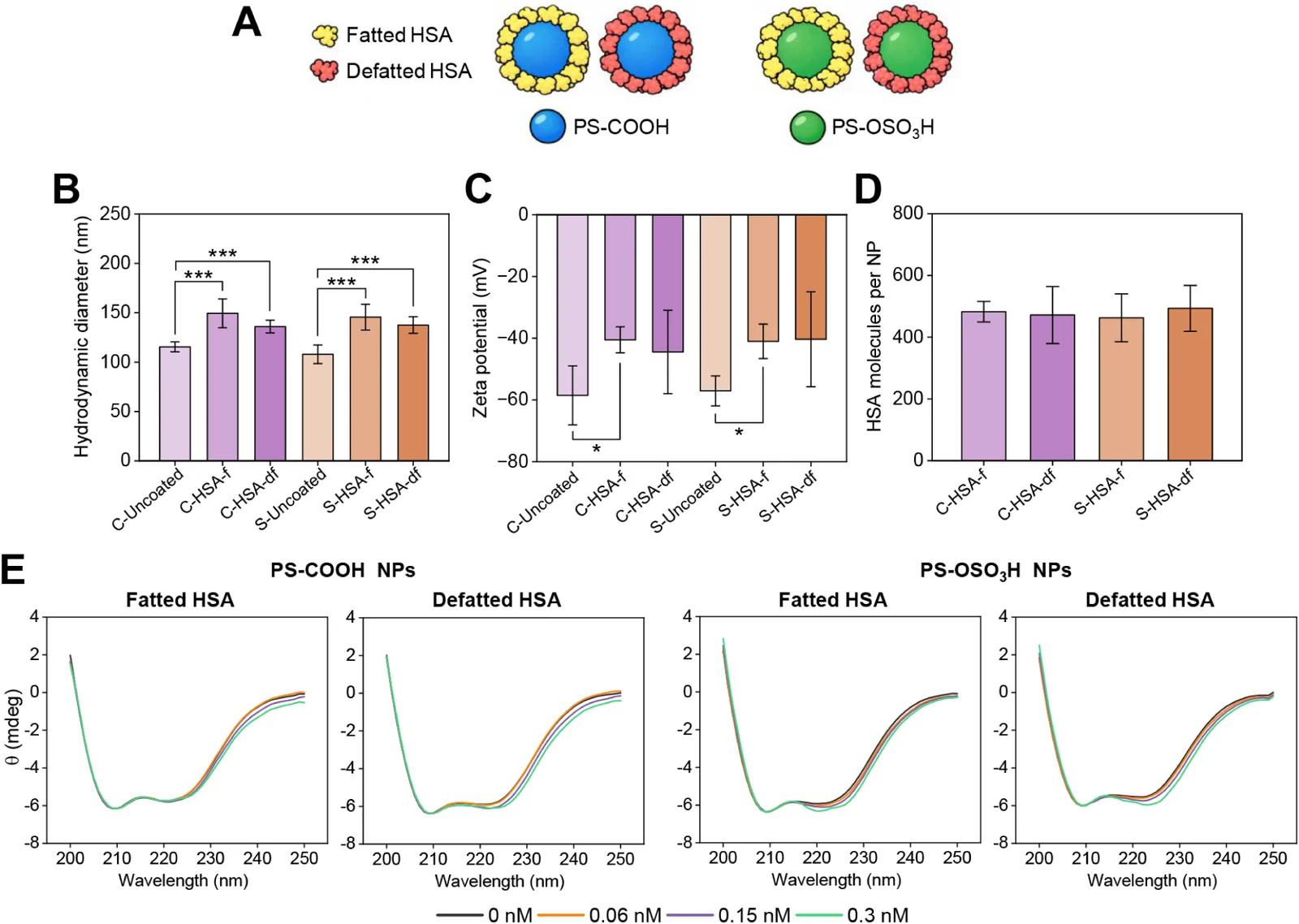
HSA precoating and physicochemical characterization of NPs. (A) Schematic of hydrophilic (PS-COOH) and hydrophobic (PS-OSO_3_H) polystyrene NPs precoated with fatted or defatted HSA (not to scale). (B) Hydrodynamic diameter determined by DLS. (C) Surface charge assessed by zeta potential measurements. (D) Estimated number of bound HSA molecules per NP from micro-BCA protein quantification. Data in (B–D) represent the mean ± SD of three to six independent replicates. Statistical analysis was performed using a two-sided *t* test. (E) Far-UV CD spectra of HSA in the presence of increasing NP concentrations (see legend). The spectra were normalized at 208 nm.

We also used circular dichroism (CD) spectroscopy to evaluate conformational changes in HSA upon adsorption (Figure 1E). The spectra were normalized at 208 nm to facilitate comparison, revealing three main trends. First, PS-COOH induced more noticeable structural changes in HSA-df than in HSA-f, consistent with the greater resistance of fatty acid-bound HSA to structural perturbation. Second, PS-OSO_3_H induced similar structural changes in both HSA variants. Third, PS-OSO_3_H induced more noticeable structural changes than PS-COOH in HSA-f, consistent with the greater tendency of hydrophobic surfaces to induce protein structural perturbation upon adsorption. We point out that the observed spectral changes were relatively modest and that a contribution from light-scattering artifacts cannot be completely excluded, as is typical for NP-containing samples. Nevertheless, because all samples were analyzed under the same experimental conditions, the spectra can still be used for relative comparisons between HSA variants and NP formulations.

We next confirmed that the NPs remained colloidally stable in biological fluid. Specifically, NPs were incubated in cell culture medium or human plasma and their stability was monitored in situ by turbidimetry. The results revealed no evidence of aggregation (Suppl. Figure S1). Finally, we evaluated the stability of the HSA precoating in biological fluid. For this, HSA was labeled with a fluorescent probe and used to prepare HSA-precoated NPs. These particles were then incubated in either buffer or human plasma, and fluorescence measurements of the supernatant after centrifugation were used to assess potential detachment of HSA. The results indicated that the HSA precoating was largely stable and remained bound even in human plasma (Suppl. Figure S2). This observation agrees with previous reports showing that preformed coronas can persist after NPs are returned to plasma.^24,25^ However, because our method provides only an approximate estimate, a minor exchange of HSA with plasma proteins cannot be excluded. Accordingly, direct covalent conjugation of HSA onto the NP surface represents a potential strategy to further enhance coating stability.^27^

### Proteomics Profiling of Adsorbed Plasma Proteins

It is now well established that NPs designed for stealth behavior can still form a hard protein corona in biological fluids.^52,53^ To evaluate this in our system, HSA-precoated NPs were incubated in 100% human plasma for 2 h, followed by centrifugation and extensive washing to remove excess and loosely bound plasma proteins. Uncoated NPs were subjected to the same treatment for comparison. These exposure conditions were selected to match those used in the whole-blood experiments (see below). The recovered surface-associated proteins, comprising both the adsorbed protein layer and the precoated HSA, were characterized by SDS-PAGE (Figure 2). Interestingly, among the HSA-precoated NPs, adsorption profiles differed between NP surface chemistries but were comparable across the two HSA variants.

**Figure 2:**
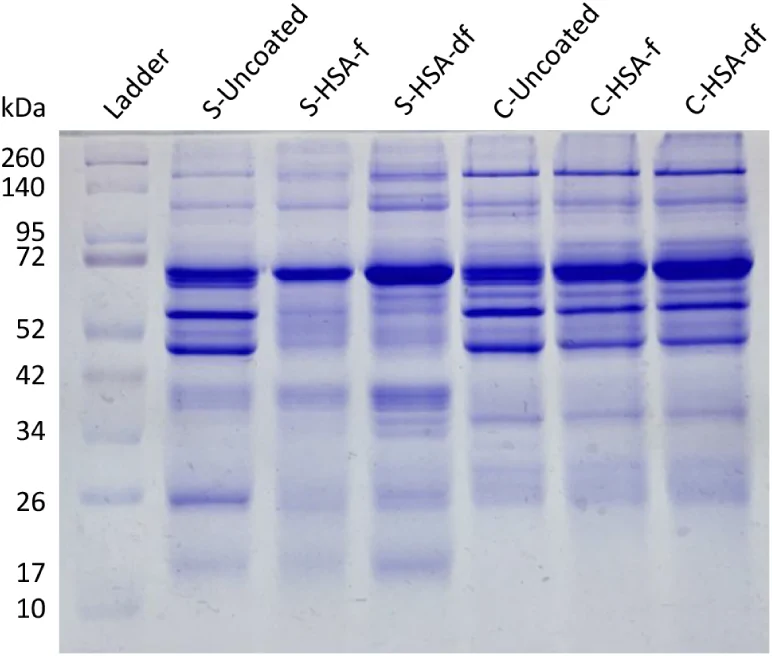
SDS–PAGE characterization of protein coronas formed on uncoated and HSA-precoated NPs.

Next, we used liquid chromatography–tandem mass spectrometry (LC–MS/MS) to identify individual proteins within the corona and quantify their relative abundances. The proteomic analysis yielded 300 protein identifications per sample, each assigned a relative protein abundance (RPA) value. Plotting the cumulative RPA of sequentially ranked proteins revealed that, across all coronas, the top 10 proteins accounted for nearly 80% of the total RPA, while the top 50 accounted for over 95% (Figure 3A). Moreover, UpSet plot analysis revealed that 180 proteins were shared across all six NP formulations, whereas virtually no proteins were uniquely associated with HSA-precoated NPs (at most, 3 unique proteins were detected for S-HSA-df) (Figure 3B). We then applied principal component analysis (PCA) to evaluate global differences in protein corona composition among the NPs (Figure 3C). The PCA revealed distinct clustering driven by NP surface chemistry, while no clear separation was observed based on the HSA variant. Protein corona profiles were further visualized using heat maps, which similarly showed clustering dominated by NP surface chemistry (Figure 3D). Importantly, both PCA and heat map analyses demonstrated strong consistency across the triplicate measurements.

**Figure 3:**
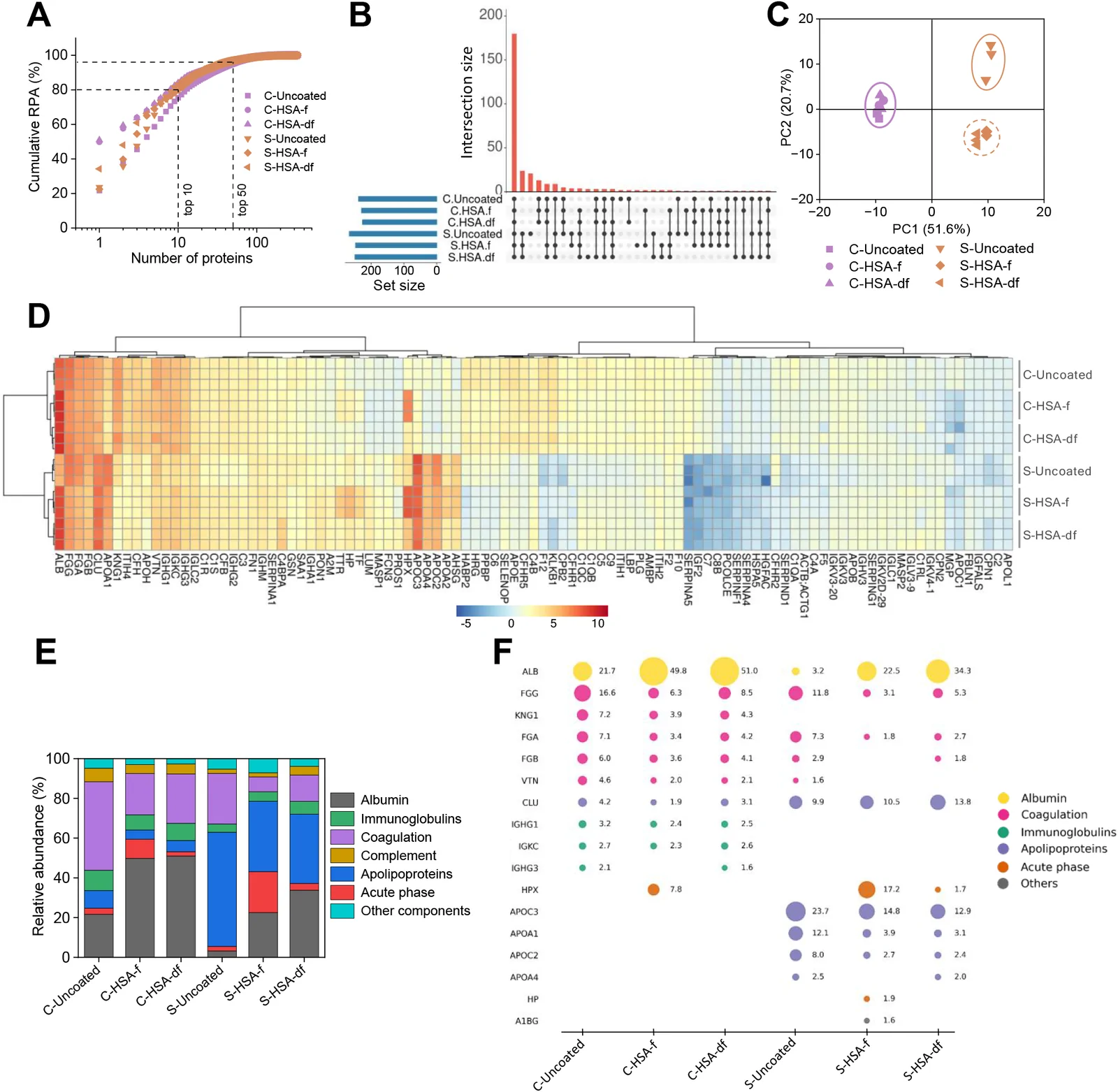
Proteomic characterization of plasma protein coronas formed on uncoated and HSA-precoated NPs. (A) Cumulative RPA of sequentially ranked corona proteins. (B) UpSet plot showing the overlap of corona proteins across NP formulations. (C) PCA of protein corona composition. (D) Hierarchically clustered heat maps of protein corona composition based on log2-transformed intensity values; only the 100 most abundant proteins are shown. (E) Functional classification of identified corona proteins according to their primary physiological roles in blood. (F) Top 10 most abundant corona proteins for each NP formulation and their corresponding RPA.

To further interpret the data, we subsequently categorized the identified proteins based on their primary physiological roles in the blood (Figure 3E). Relative to C-Uncoated and S-Uncoated, HSA-precoated NPs exhibited the expected increase in HSA content. Among the HSA-precoated formulations, S-HSA-f and S-HSA-df showed lower HSA and coagulation factor levels than C-HSA-f and C-HSA-df, which was accompanied by a compensatory increase in apolipoproteins. Importantly, complement proteins and immunoglobulins combined accounted for only 7–14% of the corona composition on HSA-precoated NPs, suggesting a reduced opsonization potential.^54^ To further characterize formulation-dependent differences in corona composition, we identified the 10 most abundant proteins associated with each NP (Figure 3F) and generated volcano plots to identify statistically significant changes in protein RPAs between HSA-precoated NP formulations (Suppl. Figure S3).

Collectively, our results showed an overall enrichment of HSA and depletion of opsonins (immunoglobulins and complement proteins) across all HSA-precoated NPs. In addition, the data suggested a more favorable corona profile for S-HSA-f and S-HSA-df compared with C-HSA-f and C-HSA-df. This distinction is mainly reflected by higher levels of apolipoproteins and lower levels of immunoglobulins and coagulation factors in the former coronas, both globally and within the top 10 proteins. Specifically, enrichment of apolipoproteins such as APOA1, APOA2, and clusterin may confer dysopsonin-like properties,^26,52,55^ whereas reduced levels of immunoglobulins and fibrinogen may decrease immune cell recognition as well as inflammatory responses and NP aggregation.^56,57^

### General Outline of Blood Compatibility Studies

After preparing and characterizing the different HSA-precoated NPs, we evaluated their hematological and immunotoxic effects, with the corresponding uncoated NPs included for comparison. The assessment focused on key parameters of NP-induced toxicity: hemolysis; thrombogenic potential, including plasma coagulation, platelet activation, and leukocyte procoagulant activity; complement activation; leukocyte cytotoxicity; and various aspects of leukocyte function, including phagocytosis, activation, proliferation, and cytokine release.^17,58,59^ Collectively, these assessments adhere to test criteria established by the ISO 10993 series for the biological evaluation of medical devices and by the National Cancer Institute Nanotechnology Characterization Laboratory.^17,60–62^

To preserve the physiological interactions and natural cross-talk among cellular and plasma components, we implemented an ex vivo human whole-blood model using the Chandler loop system (Figure 4A).^8,48,63–65^ NPs were incubated with blood for 2 h in the Chandler loop system, after which hemostasis and inflammation biomarkers were analyzed in plasma and blood cells (Figure 4A). Additional details are provided in the Materials and Methods section and Suppl. Figure S4. In addition to whole-blood assays, we conducted complementary experiments with human plasma (Figure 4B) and peripheral blood mononuclear cells (PBMCs) (Figure 4C). The NP concentration (40 μg mL^–1^) used in the experiments was guided by prior mouse studies in which polymeric NPs were administered intravenously at doses ranging from 3 to 140 mg kg^–1^.^66–71^ This range was then translated into an equivalent in vitro NP concentration using a published scaling formula.^61^ This estimate represents a conservative upper bound, as it assumes that the entire injected dose is confined to the blood compartment.

**Figure 4:**
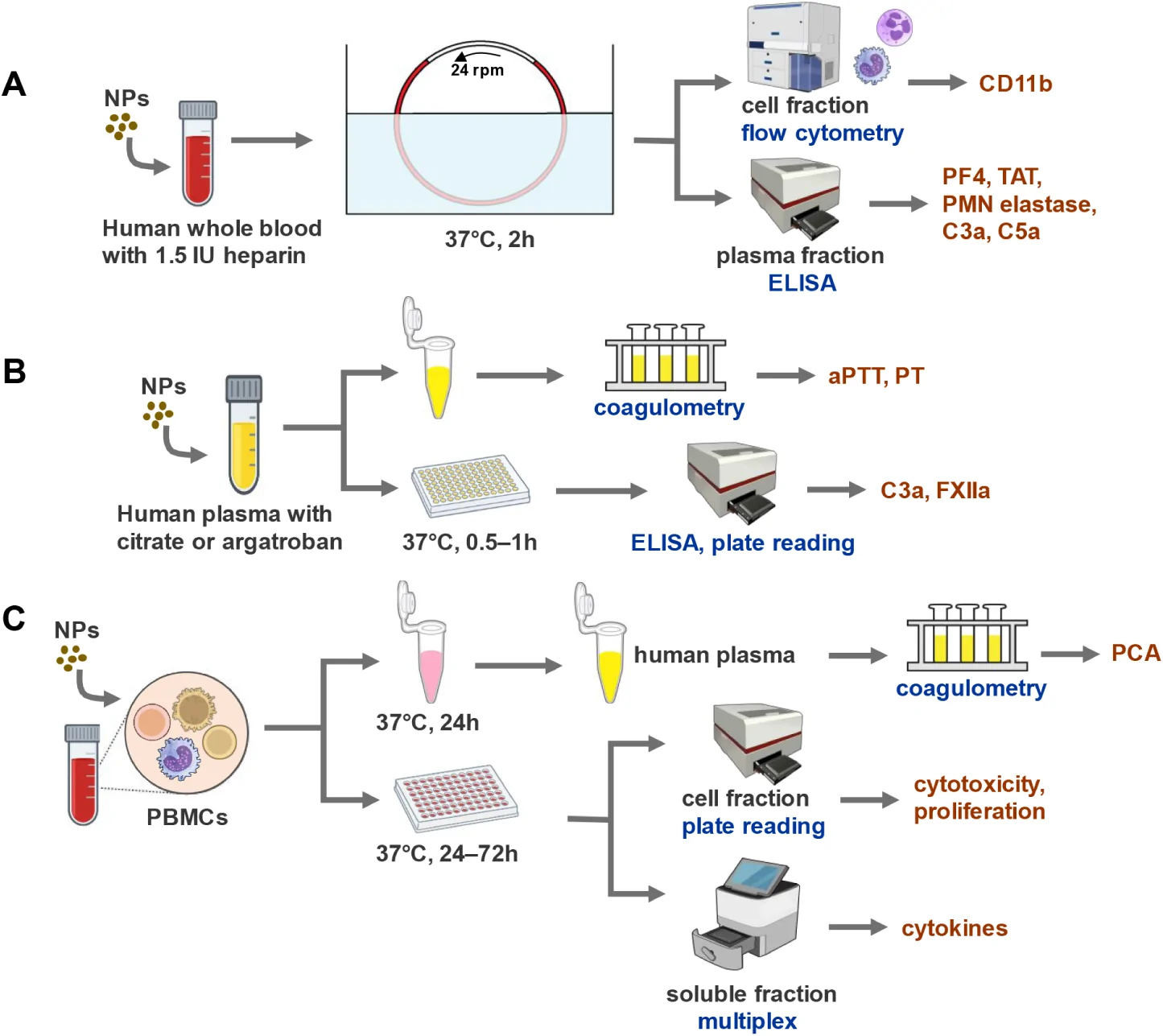
Schematic representation of blood compatibility assays. (A) Ex vivo human whole blood model (Chandler loop). NPs are dispersed in whole blood containing a low dose of heparin (1.5 IU heparin mL^–1^) as an anticoagulant. The blood is loaded into polymer tubing and rotated for 2 h at 37 °C. Plasma and blood cells are then analyzed for hemostatic and inflammatory biomarkers. (B) NPs are incubated in human plasma anticoagulated with sodium citrate or argatroban for 0.5 to 1 h to assess their effects on the coagulation cascade and complement system. (C) PBMCs are exposed to NPs in cRPMI medium for 24–72 h to evaluate cytotoxicity, leukocyte proliferation, leukocyte PCA, and cytokine release. Additional experiments using blood and platelet-rich plasma were conducted to further investigate NP uptake and platelet aggregation (not shown; see text for details). Some illustrations were adapted from the NIAID NIH BioArt source (https://bioart.niaid.nih.gov).

### NP Uptake by Leukocytes and Cytotoxicity

NP uptake by phagocytic blood cells is one of the most widely used readouts to assess NP stealth properties. To evaluate this, we incubated fluorescently labeled NPs with whole blood and analyzed NP uptake by monocytes and granulocytes using flow cytometry (Figure 5). Uptake of C-Uncoated and S-Uncoated NPs reached 32–53% of cells, whereas uptake of all HSA-precoated NPs remained low (<10%). Notably, S-HSA-f and S-HSA-df showed particularly low uptake, with only ∼2% of cells being NP-positive. These results confirm that HSA precoating is an effective strategy to delay NP uptake by phagocytic blood cells. To evaluate NP-induced cytotoxicity, we exposed isolated PBMCs to NPs for 24 h and analyzed Annexin V staining by flow cytometry. No significant cytotoxicity was observed under these conditions (Suppl. Figure S5).

**Figure 5:**
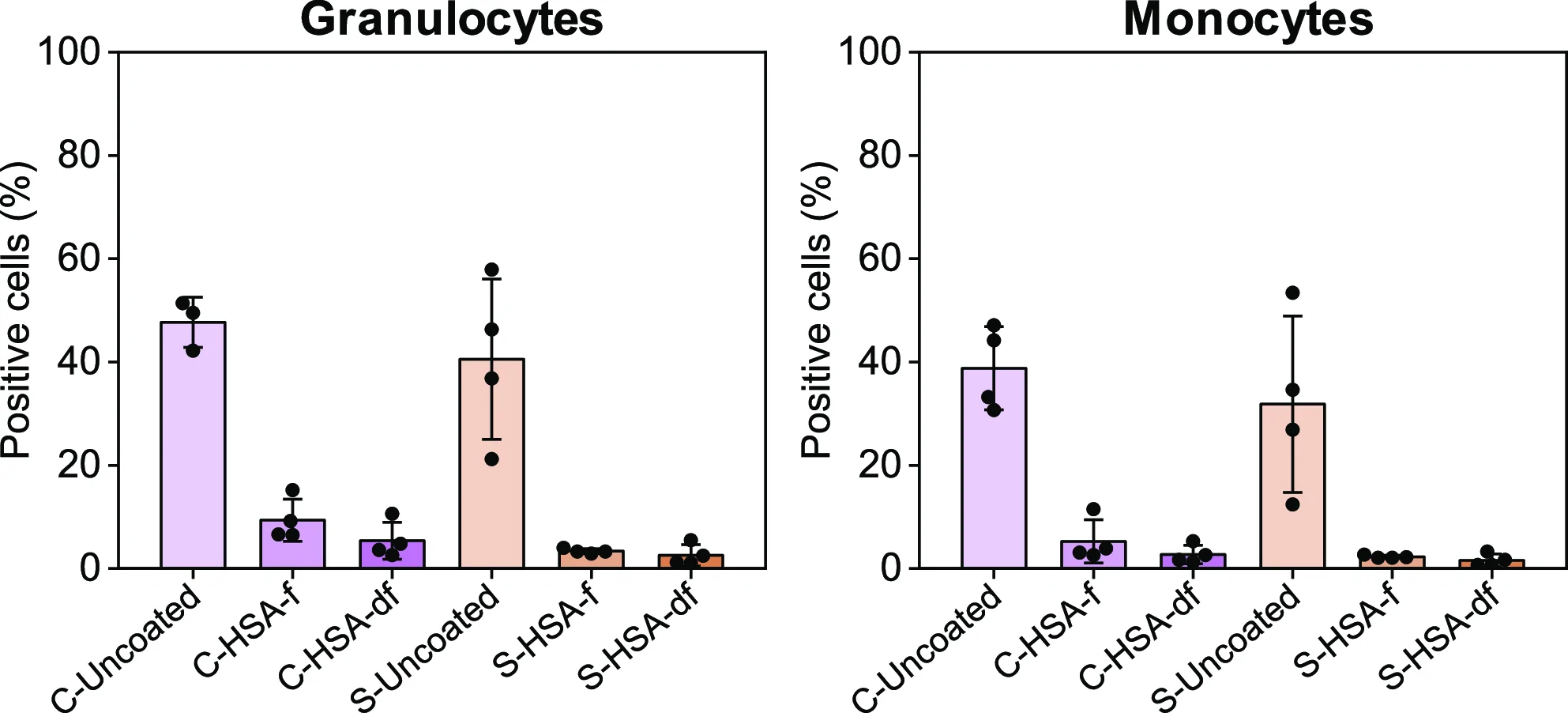
NP uptake by leukocytes in whole blood. Whole blood was incubated with fluorescently labeled NPs in 96-well plates for 2 h at 37 °C (*n* = 4 donors). Internalization by granulocytes and monocytes was quantified by flow cytometry and is represented as the percentage of NP-positive cells.

### Hematological Effects

Hemolysis represents one of the fundamental assays used to evaluate the safety and blood compatibility of NPs.^72^ To assess this, NPs were circulated with whole blood in the Chandler loop system, after which plasma-free hemoglobin was quantified by absorbance. The percentage of hemolysis was 3.3% for S-Uncoated and below 2–3% for all other formulations (Suppl. Figure S6A). Hence, with the exception of S-Uncoated, which could be considered moderately hemolytic, all other NPs were nonhemolytic.^17,61^

Platelets play a key role in hemostasis, making it essential to determine whether NPs affect their activity.^59,72^ We first assessed platelet activation in whole blood by measuring platelet factor 4 (PF4) release using enzyme-linked immunosorbent assay (ELISA). The S-Uncoated formulation induced higher PF4 levels than the control, although the difference was not statistically significant (Suppl. Figure S6B). To complement this analysis, NPs were incubated in platelet-rich plasma (PRP), and their effects on platelet activation were evaluated by light transmission aggregometry. Again, S-Uncoated showed a trend toward increased platelet activation (Suppl. Figure S6C). All other NPs did not induce platelet aggregation (Suppl. Figure S6C), and none interfered with aggregation induced by the agonist arachidonic acid (Suppl. Figure S6D).

The coagulation cascade can be inadvertently triggered by NPs, particularly those with an anionic surface, through a process known as contact activation in which factor XII (FXII) is converted to its active form (FXIIa).^73^ Unregulated NP-induced contact activation can result in severe complications, including disseminated intravascular coagulation and deep vein thrombosis. Alternatively, NP interactions with coagulation factors can lead to prolonged clotting times and cause bleeding complications. Assessing both activation and inhibition of the coagulation cascade is essential to ensure the safe clinical translation of NPs.^9,74–76^ We first evaluated NP-induced activation of the coagulation cascade in human plasma by measuring FXIIa and plasma kallikrein (PKa) generation using a chromogenic assay. C-Uncoated efficiently triggered contact activation, with C-HSA-f and C-HSA-df also showing some effect (Figure 6A). NPs were subsequently assessed in whole blood for their capacity to generate thrombin–antithrombin (TAT) complexes. In this setting, C-Uncoated did not induce thrombin generation despite its anionic surface. In contrast, both C-HSA-f and C-HSA-df showed a trend toward elevated TAT levels, although the difference was not statistically significant (Figure 6B). Interestingly, some donors exhibited markedly increased TAT levels, suggesting interindividual variability in the response to NPs. We next applied standardized activated partial thromboplastin time (aPTT) and prothrombin time (PT) assays to evaluate whether the NPs interfered with normal propagation of the coagulation cascade. Both C-Uncoated and C-HSA-f prolonged aPTT, indicating interference with downstream reactions and a delay in coagulation when this was triggered by a strong activator (Figure 6C). In contrast, none of the NP formulations affected PT (Figure 6D).

**Figure 6:**
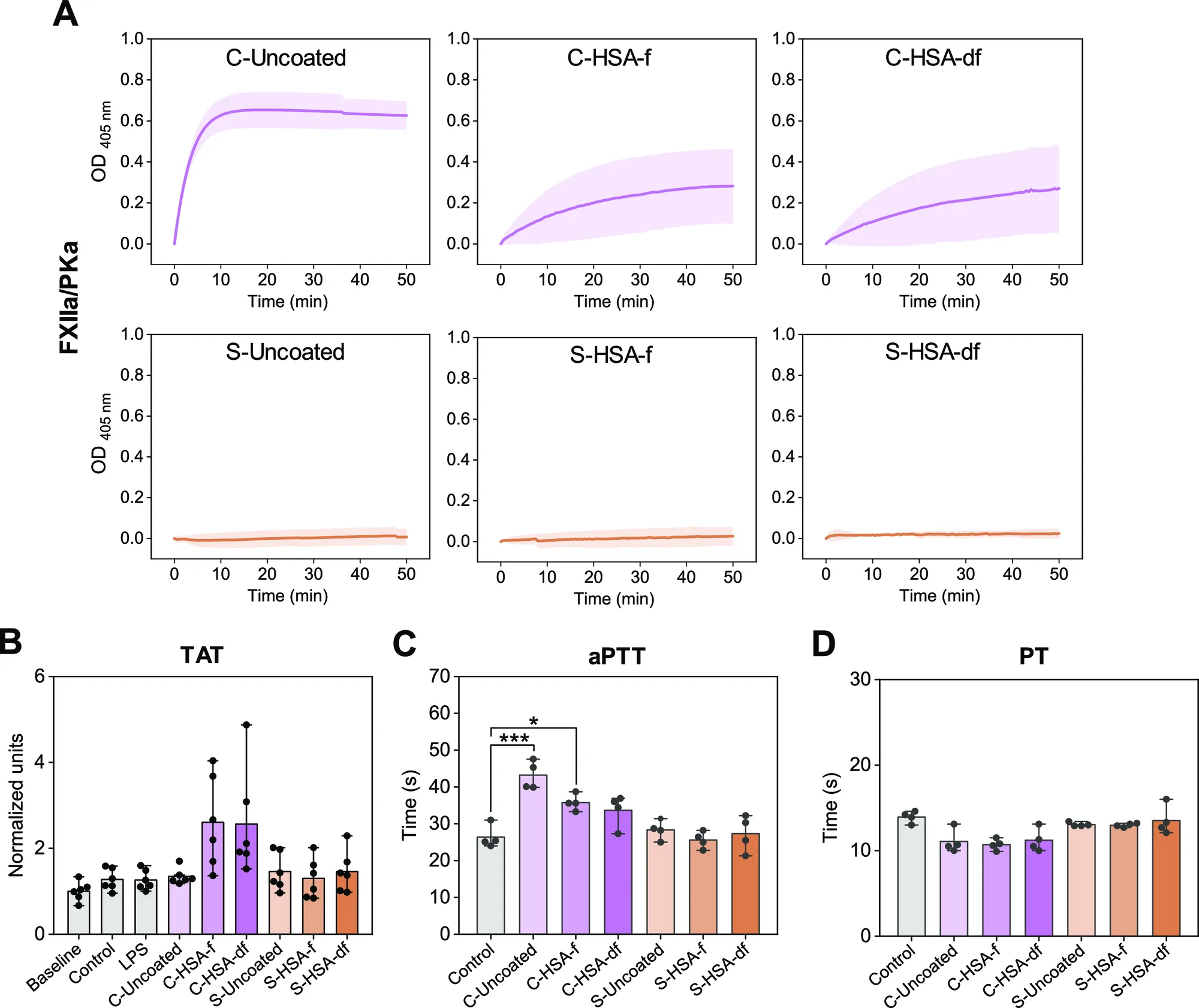
NP interference in the coagulation cascade. (A) Progress curves for FXIIa and PKa generation. Citrated human plasma was incubated with NPs for 1 h at 37 °C, followed by addition of the chromogenic substrate S-2302. Absorbance was recorded at 405 nm in a plate reader (*n* = 3). (B) TAT complex formation in whole blood. NPs were circulated with whole blood in the Chandler loop system, followed by quantification of the TAT complex in the plasma fraction using ELISA (*n* = 6). Baseline and control refer to blood without NPs before and after circulation, respectively. Results were normalized to the baseline. Statistical analysis was performed using Welch’s ANOVA followed by the Games–Howell post hoc test, which revealed no significant differences. (C, D) aPTT and PT standard assays. Citrated human plasma was incubated with NPs for 0.5 h at 37 °C, followed by addition of reagents for (C) aPTT or (D) PT. Clotting times were measured using a coagulometer (*n* = 4). Buffer without NPs served as control. Statistical analysis was performed using one-way ANOVA followed by Tukey’s post hoc test.

The complement system serves as a critical first line of defense against invading pathogens. Its activation occurs via three primary pathways: classical, alternative, and mannose-binding lectin. All three pathways converge at the cleavage of complement component C3, generating the opsonin C3b, which promotes phagocytosis, and the anaphylatoxin C3a, which triggers inflammatory responses. The cascade continues with the cleavage of C5 into C5a and C5b. Unintended complement activation by NPs can elicit immune responses of varying degrees.^11,54^ To investigate this, NPs were incubated in whole blood, and plasma levels of C3a and C5a were measured by ELISA. While C5a concentrations remained unchanged relative to the negative control, a mild increase in C3a levels was observed for C-HSA-df and S-HSA-df (Figure 7A). To verify whether this response was consistent across different experimental setups, NPs were incubated with platelet-poor plasma (PPP), and C3a levels were measured again by ELISA. Here, plasma was anticoagulated with the direct thrombin inhibitor argatroban, a method expected to better preserve complement activity than heparin.^77^ Except for S-HSA-f, all NPs showed a trend toward increased C3a levels, although differences did not reach statistical significance (Figure 7B).

**Figure 7:**
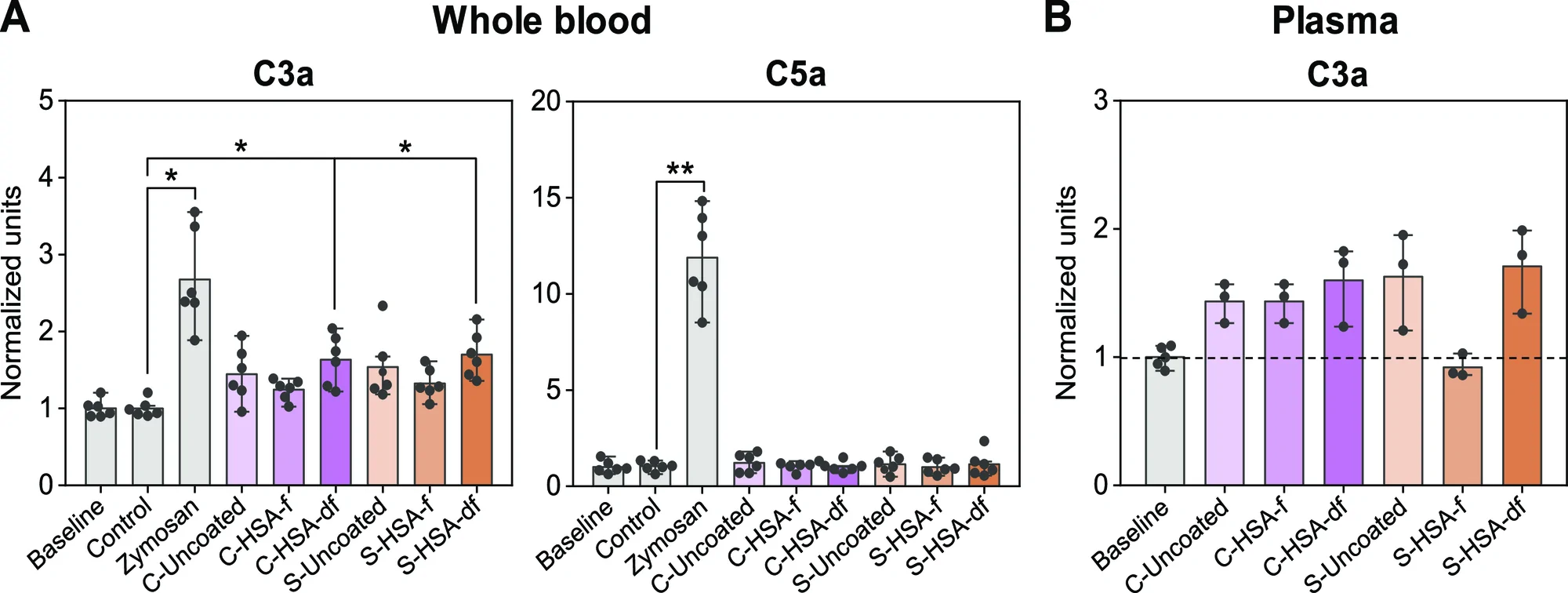
NP-induced complement activation. (A) Generation of C3a and C5a in whole blood. NPs were circulated with whole blood in the Chandler loop system, followed by quantification of C3a and C5a levels in the plasma fraction using ELISA (*n* = 6). Baseline and control refer to blood without NPs before and after circulation, respectively. Zymosan was added to plasma as a positive control. Results were normalized to the baseline. Statistical analysis was performed using Welch’s ANOVA followed by the Games–Howell post hoc test. (B) Generation of C3a in human plasma. PPP was incubated with or without NPs for 1 h at 37 °C, and C3a levels were measured using ELISA (*n* = 3). Results were normalized to the buffer control without NPs (baseline). Statistical analysis was performed using nonparametric Kruskal–Wallis test followed by Dunn’s posthoc test, which revealed no significant differences.

### Leukocyte Responses

While none of the NP formulations caused substantial cytotoxicity (Suppl. Figure S5), their interactions with leukocytes could still influence immune responses. We therefore assessed the effects of the NPs on key leukocyte end points.^78,79^ First, we evaluated leukocyte activation by exposing whole blood to NPs and measuring early activation markers, including CD11b surface expression on monocytes and neutrophils and the release of polymorphonuclear (PMN) elastase from neutrophils. The results showed that none of the NPs increased these markers (Figure 8). This lack of detectable activation likely reflects limited NP uptake and relatively low levels of C5a generation, as C5a is the more potent proinflammatory anaphylatoxin compared to C3a.

**Figure 8:**
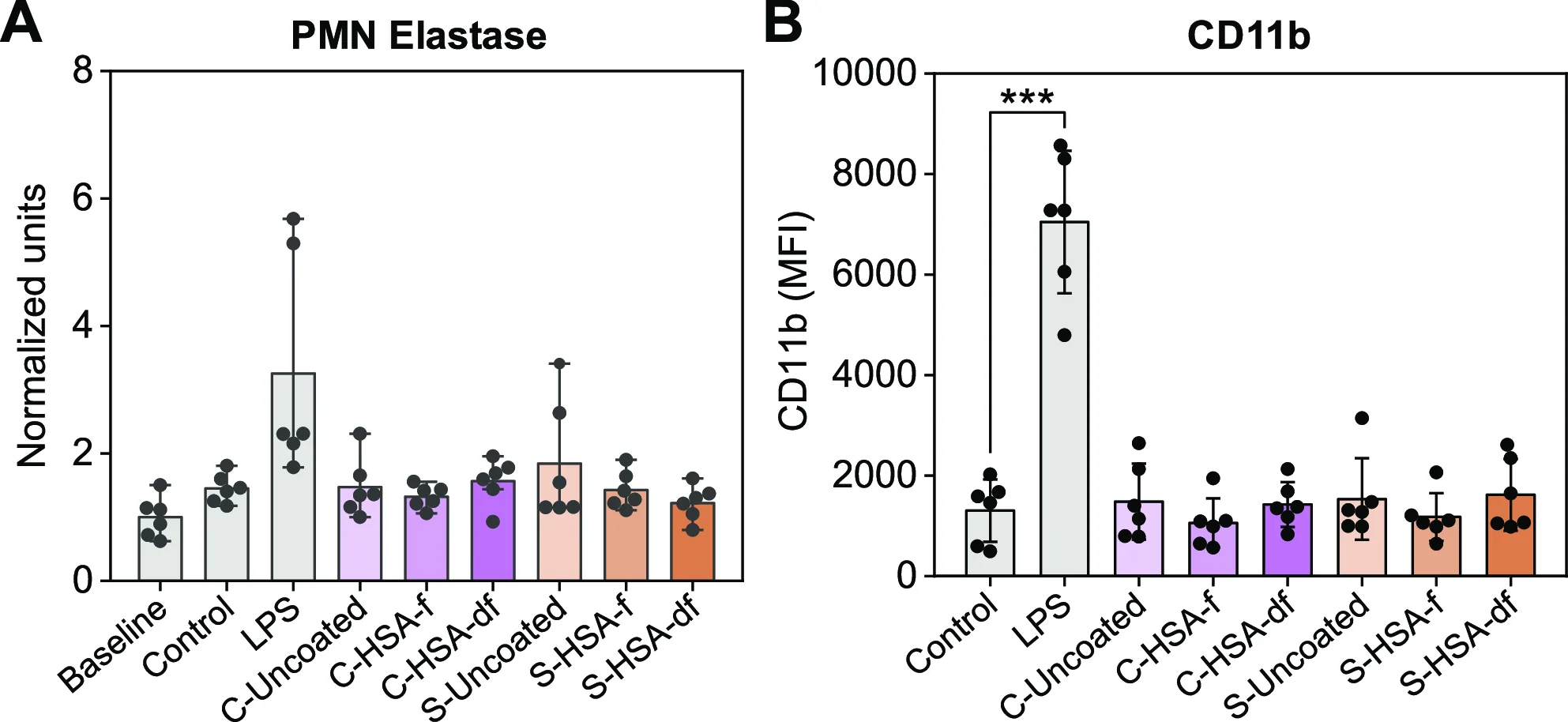
NP-induced leukocyte activation in whole blood. (A) PMN elastase release. (B) CD11b surface expression. NPs were circulated with whole blood in the Chandler loop system, followed by quantification of PMN elastase levels in plasma by ELISA and CD11b surface expression by flow cytometry (*n* = 6). Baseline and control refer to blood without NPs before and after circulation, respectively. LPS (100 ng mL^–1^) was included as a positive control. Results in (A) were normalized to baseline. Statistical analysis was performed using the nonparametric Kruskal–Wallis test followed by Dunn’s post hoc test for (A), and one-way ANOVA followed by Tukey’s post hoc test for (B); no statistically significant differences were observed.

To further assess NP–immune cell interactions, we examined cytokine release from isolated PBMCs. Cells were exposed to each NP formulation for 24 h, and cytokine levels were quantified using a multiplex-based assay. PBMCs cultured without NPs served as a control, while LPS stimulation provided a benchmark against which to compare NP-induced cytokine levels. We quantified the pro-inflammatory cytokines TNF-α, IL-6, and IL-1α, the chemokine IL-8, and the anti-inflammatory cytokine IL-10 (Figure 9). With the exception of S-HSA-df, all NPs increased IL-8 to levels comparable to LPS. In addition, C-HSA-f also led to elevated IL-10 levels. By contrast, NP exposure did not significantly alter the levels of pro-inflammatory cytokines relative to the negative control.

**Figure 9:**
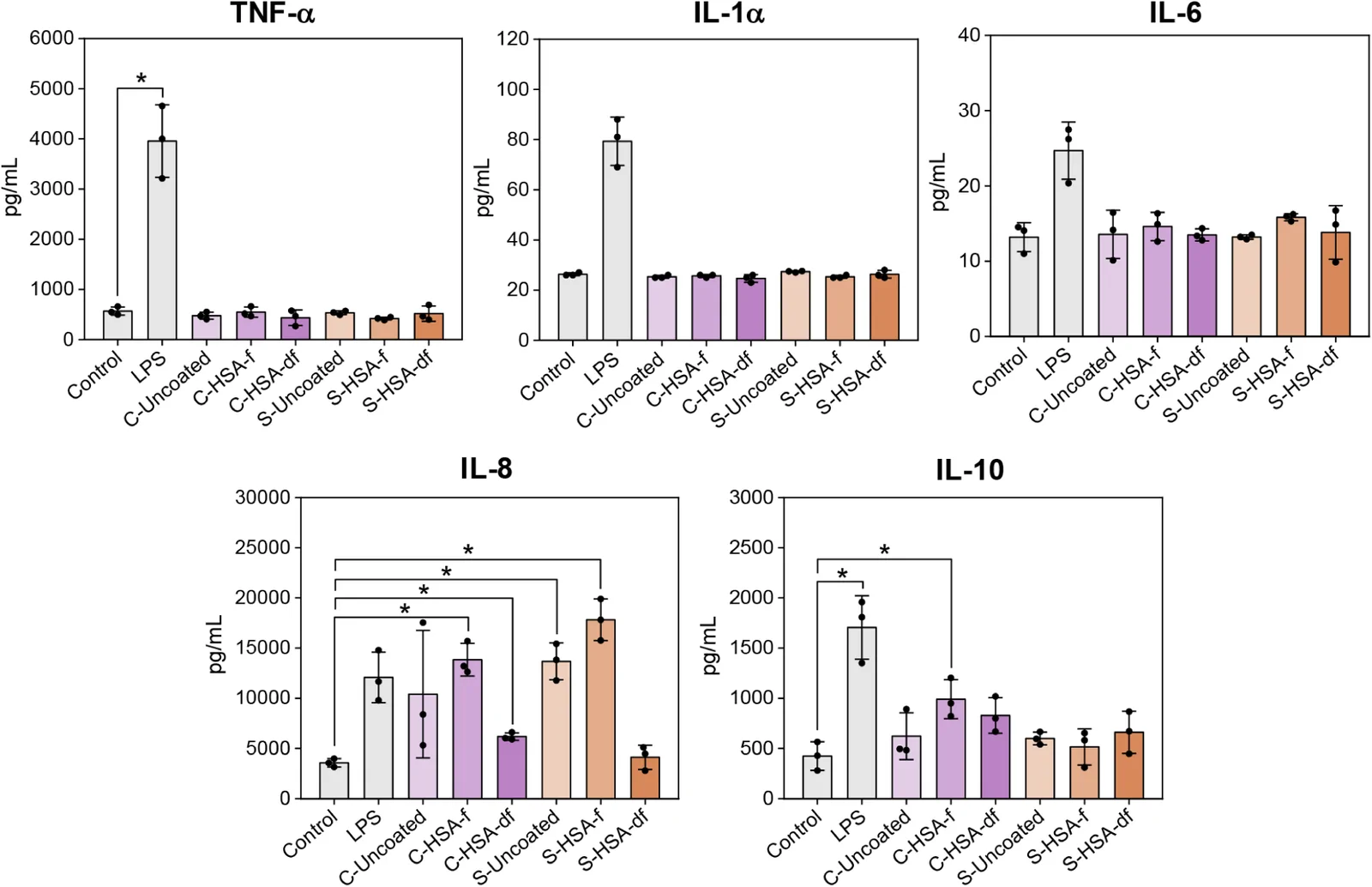
NP-induced cytokine release. PBMCs in cRPMI were incubated with or without NPs for 24 h, followed by cytokine quantification. PBMCs containing LPS (100 ng mL^–1^) were used as the positive control (*n* = 3). Statistical analyses were performed using Welch’s ANOVA followed by the Games–Howell post hoc test or using one-way ANOVA followed by Tukey’s post hoc test.

Leukocytes can contribute to thrombogenicity by activating the plasma coagulation cascade. This occurs through surface expression of the procoagulant activity (PCA) complex, composed of tissue factor and phosphatidylserine.^79^ To evaluate whether the test NPs induced leukocyte PCA, we exposed PBMCs to NPs for 24 h, followed by incubation of plasma with the treated cells and measurement of plasma clotting times. All NPs with carboxyl-modified surfaces (C-Uncoated, C-HSA-f, and C-HSA-df) significantly increased leukocyte PCA, as evidenced by the marked reduction in plasma clotting times compared to the negative control (Figure 10A). In contrast, S-Uncoated, S-HSA-f, and S-HSA-df did not induce leukocyte PCA.

**Figure 10:**
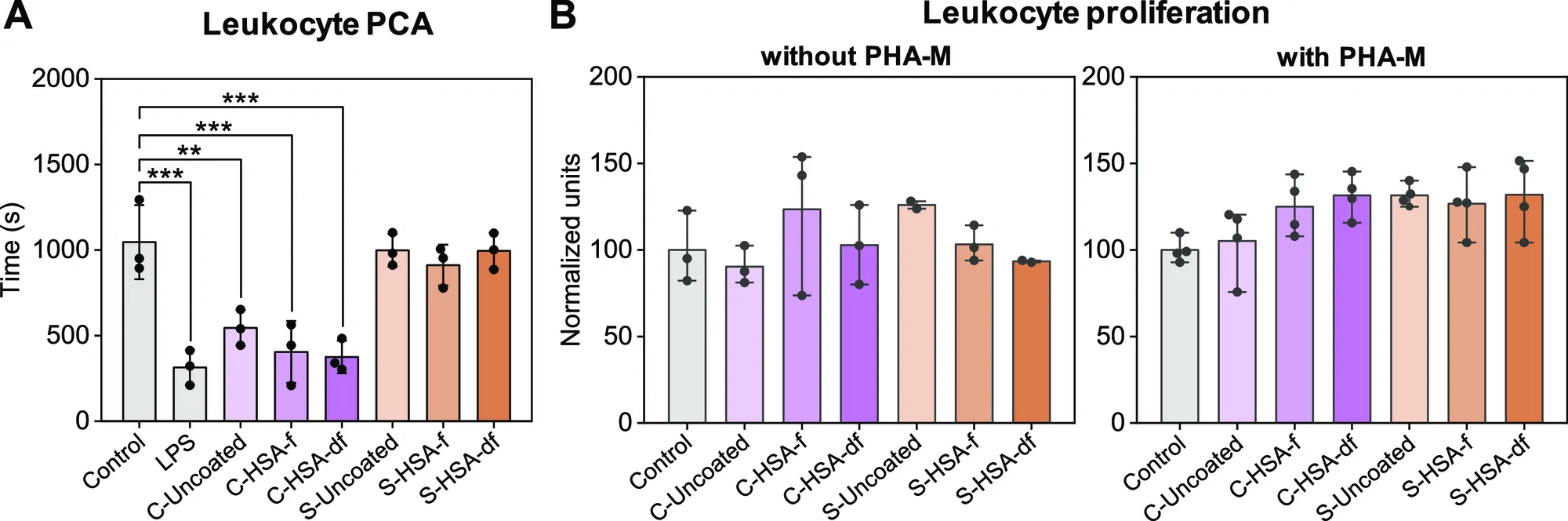
Leukocyte PCA and leukocyte proliferation. (A) NP-induced leukocyte PCA. PBMCs in cRPMI were incubated with or without NPs for 24 h. PBMCs containing LPS (100 ng mL^–1^) were used as the positive control. After incubation, cells were washed and resuspended in human plasma, and clotting times were measured using a coagulometer (*n* = 3). (B) NP regulation of leukocyte proliferation. PBMCs in cRPMI were cultured with NPs for 72 h, with or without PHA-M (10 μg mL^–1^). Samples without NPs served as the negative control. Leukocyte proliferation was measured by MTT assay (*n* = 3–4). Statistical analyses were performed using one-way ANOVA followed by Tukey’s post hoc test.

To further investigate the potential impact of the NPs on immune cell function, we examined their ability to induce lymphocyte proliferation or suppress mitogen-driven activation.^17,79^ For this purpose, PBMCs were exposed to NPs for 72 h, either in the absence or presence of the mitogen phytohemagglutinin (PHA-M), and leukocyte proliferation was quantified using the MTT assay. We found that NPs alone did not stimulate leukocyte proliferation compared with the no-NP control (Figure 10B, left panel). Moreover, in the presence of PHA-M, none of the NPs suppressed proliferation relative to the no-NP control (Figure 10B, right panel).

## Summary and Discussion

We prepared four distinct HSA-precoated polystyrene NPs by varying both the NP surface chemistry (hydrophilic/hydrophobic) and the albumin type (fatted/defatted). Interestingly, our CD results suggested small structural changes that depended on both the HSA formulation and NP core chemistry, indicating that both factors can influence the structural state of the HSA precoating. However, a more detailed characterization of these structural differences will require complementary analytical techniques. We also found that the HSA coatings were stable under plasma exposure, with no detectable detachment.

Upon exposure to human plasma, the HSA-precoated NPs acquired a hard protein corona comprising diverse plasma proteins. Given a fixed NP surface chemistry, coronas formed on fatted and defatted HSA coatings were broadly similar. Still, the coronas were not identical, and differences in the abundance of a limited number of proteins were detected. This observation underscores the high sensitivity of protein corona formation to relatively minor differences in the underlying HSA precoating layer. In contrast, coronas formed on HSA-precoated PS-COOH and PS-OSO_3_H exhibited more distinct profiles. While protein coronas formed on all HSA-precoated NPs displayed a generally favorable profile, those formed on S-HSA-f and S-HSA-df exhibited the most favorable composition, as discussed earlier.

As a next step, we evaluated NP blood compatibility using a series of human-based assays. All HSA-precoated NPs were safe with respect to acute hematological end points, showing no evidence of hemolysis, fibrin clot formation, or platelet aggregation. In addition, HSA-precoated NPs exhibited low uptake by phagocytic blood cells, with S-HSA-f and S-HSA-df being internalized by only ∼2% of cells. The HSA-precoated NPs also did not induce cytotoxicity, leukocyte activation, or alterations in leukocyte proliferation. However, other assays revealed that the NPs could still elicit unintended biological effects. In particular, both C-HSA-df and S-HSA-df induced mild complement activation, while C-HSA-f and C-HSA-df showed prothrombotic tendencies, as indicated by trends toward increased TAT complex formation, FXIIa/PKa generation, and leukocyte PCA activation. This observation reinforces that reliance on limited readouts, such as hemolysis and NP uptake by phagocytic blood cells, as commonly used in the literature, is insufficient for accurately assessing NP blood compatibility.

Among the four HSA-precoated formulations, only S-HSA-f demonstrated high blood compatibility across the evaluated assays. This behavior stems not from resistance to plasma protein corona formation, but rather from the specific properties of the adsorbed corona. A similar interpretation extends to PEG, other so-called stealth polymers, and alternative surface chemistries reported to confer stealth behavior.^52,80^ Notably, despite its overall hemocompatibility, S-HSA-f induced selective chemokine (IL-8) signaling in PBMCs after 24 h. IL-8 is an important mediator of inflammatory cell recruitment, and its induction by S-HSA-f may therefore have biological consequences. Nevertheless, the IL-8 increase was not accompanied by measurable leukocyte activation or immunotoxicity in the assays performed here. Together, these findings suggest that HSA precoating may influence selective immune responses without measurably compromising hemocompatibility, warranting further investigation into the underlying mechanisms and biological significance of this response.

At this point, it is useful to examine how the hard protein corona composition relates to the biological outcomes observed in our system. However, it is important to first recognize that, beyond composition, the structural and functional states of proteins within the hard corona can also influence NP bioresponses. In addition, the soft corona, comprising transiently bound proteins, can also contribute to these bioresponses.^81,82^ Despite these limitations, hard corona composition remains widely used as a first approximation for understanding NP bioresponses. Within this framework, we observed corona profiles enriched in dysopsonins and depleted in major opsonins, which likely accounts for the minimal uptake of HSA-precoated NPs by blood phagocytes. Moreover, C-HSA-f and C-HSA-df were enriched in proteins associated with contact activation of the coagulation cascade, particularly FXII and PK, which was consistent with the increased prothrombotic tendencies observed for these formulations. In contrast, no clear relationship was found between C3a generation by C-HSA-df and S-HSA-df and the relative abundance of C3-derived species detected in the corresponding coronas. This latter finding illustrates that hard corona composition is informative but not sufficient to predict all NP bioresponses, reinforcing the importance of direct and multiparametric blood compatibility assessments.

## Conclusions

In this study, we evaluated HSA precoating as a strategy to generate blood-compatible NPs. To minimize species-dependent effects, experiments were performed using human-derived models, including ex vivo whole-blood assays. All tested HSA-precoated NPs formed favorable protein coronas enriched in dysopsonins and depleted in major opsonins. Consistent with this overall corona composition, all formulations exhibited low uptake by phagocytic blood cells. Nevertheless, differences in hematological and immunotoxic responses were observed among formulations, with only fatted HSA on hydrophobic NPs showing consistently favorable outcomes across the evaluated assays. Collectively, these findings demonstrate that the biological performance of albumin-coated NPs is not determined by albumin precoating alone but depends on the interplay between NP surface chemistry and the fatty acid loading of albumin. This work therefore provides important considerations for the rational design and evaluation of albumin-based nanomedicines.

## Materials and Methods

### Reagents

Fatted HSA, defatted HSA, Atto 488 NHS, and PHA-M were from Sigma-Aldrich (São Paulo, Brazil). Carboxyl- and sulfate-modified nonfluorescent polystyrene NPs of 100 nm in diameter were from Magsphere Inc. (California, USA), while corresponding fluorescently labeled NPs were from Thermo Fisher (Massachusetts, USA). RapiGest SF was from Waters (Massachusetts, USA). Micro-BCA protein quantification kit, Limulus amebocyte lysate assay kit, the various ELISA kits, and anti-CD11b-PE were from Thermo Fisher. BD Lysing Buffer was from BD Biosciences (São Paulo, Brazil). Ficoll-Paque Plus was from Cytiva (Massachusetts, USA).

### Buffers and Cellular Media

Phosphate buffer (20 mM, pH 7.4) solutions with or without 150 mM NaCl (PBS and PB, respectively) and HEPES buffer (20 mM, pH 7.4, 150 mM NaCl) were used in various experiments as detailed below. HEPES–Tyrode (5 mM HEPES, 137 mM NaCl, 2.9 mM KCl, 12 mM NaHCO_3_, 5 mM C_6_H_12_O_6_, 1 mM CaCl_2_, 1 mM MgCl_2_, pH 7.4) was used for platelet aggregation experiments. PBMCs were maintained in complete RPMI medium (cRPMI), consisting of RPMI 1640 supplemented with 10% fetal bovine serum (FBS).

### NP Preparation and Characterization

NPs (400 μg mL^–1^) were incubated in PB with either fatted or defatted HSA (10 mg mL^–1^) for 24 h at 4 °C. HSA-precoated NPs were collected after three cycles of centrifugation and washing, followed by resuspension in PB. Both uncoated and HSA-precoated NPs (4 μg mL^–1^) were characterized for size and surface charge by DLS and zeta potential measurements (pH 7.4, 25 °C) on a Zetasizer Nano ZS analyzer (Malvern Instruments, UK). To estimate the amount of HSA adsorbed per NP, HSA-precoated NP pellets were resuspended in 50 mM NH_4_HCO_3_ buffer containing 0.2% RapiGest and incubated at 90 °C for 15 min to detach proteins from the NP surface. After centrifugation, the supernatant was collected, and protein concentrations were quantified using the Micro-BCA protein assay kit. Binding stoichiometry was calculated by dividing the measured protein amount (in nM) by the NP concentration (in nM). The theoretical binding stoichiometry was estimated by dividing the total NP surface area by the albumin cross-sectional area, yielding ∼800 albumin molecules per NP. To evaluate potential HSA structural changes upon adsorption, HSA variants (0.5 μM) were titrated with NPs (0.06, 0.15, and 0.3 nM) in PBS, and far-UV CD spectra were recorded using a Chirascan Plus spectropolarimeter (Applied Photophysics, UK). Spectra were acquired from 200 to 250 nm using a bandwidth of 2 nm and a step size of 1 nm. For each NP concentration, a corresponding background spectrum of NPs dispersed in PBS was recorded under identical conditions and subtracted from the sample spectrum. The maximum NP concentration was selected to keep the high-tension voltage below ∼800 V, thus minimizing detector noise. For each sample, eight scans were collected, averaged, and smoothed using a Savitzky–Golay filter. To assess HSA stability on the NP surface, NPs were coated with fluorescently labeled HSA and evaluated as described in the Supporting Information (Suppl. Figure S2). Finally, NP stocks were tested for endotoxin contamination using the Limulus amebocyte lysate assay to ensure that any observed immunological responses were not attributable to endotoxins. After adjusting for NP dilution to match experimental concentrations, endotoxin levels were 0.02 EU/mL for C-HSA-df and S-HSA-df, and 0.001–0.004 EU/mL for all other formulations, representing low contamination levels.

### Biological Samples

All experiments involving human subjects were approved by the Research Ethics Committees of the Universidade Federal de São Paulo (UNIFESP) and the Centro Universitário Faculdade de Medicina do ABC (FMABC) (approval #78406224.0.0000.5505). Informed consent was obtained from all participants prior to their inclusion in the study. Approximately 2 mL of blood were collected from normal volunteers and anticoagulated with 1.5 IU heparin mL^–1^ for use in Chandler loop experiments and NP uptake studies as described below. This moderate heparin dose maintained anticoagulation throughout the experiments while preserving complement system functionality. Blood samples were used within a maximum of 5 h from collection to the completion of NP experiments. Additionally, blood samples anticoagulated with sodium citrate were collected for PBMC isolation, which was performed using Ficoll-Paque Plus density gradient according to the manufacturer’s instruction. Human PPP, anticoagulated with sodium citrate, was obtained from the Charitable Association of Blood Collection (COLSAN, São Paulo, SP). The plasma was pooled from three individual donors, aliquoted, and stored at −80 °C until use. Each aliquot was thawed once for use in experiments, with any unused plasma discarded. The plasma samples were subsequently used to investigate protein corona formation by mass spectrometry and to evaluate coagulation and complement systems, as outlined below. Finally, platelet concentrates in plasma (PRP) were obtained from COLSAN for platelet aggregation experiments, as described below.

### LC-MS/MS Characterization

Uncoated and HSA-precoated NPs (40 μg mL^–1^) were incubated in 100% PPP at 37 °C for 2 h in triplicate. Excess and loosely bound proteins were then removed by five cycles of centrifugation and washing. After the final wash, the adsorbed protein corona was stripped from the NP surface as described above. Proteins were reduced using DTT (5 mM), incubated for 30 min at 65 °C, and alkylated with IAA (15 mM) at room temperature in the dark for 30 min. Trypsin (1:100 w/w) digestion was performed overnight at 37 °C. Digestion was stopped with 5% TFA, and samples were desalted with StageTips.^83,84^ Samples were then resuspended in 0.1% formic acid (FA), and analyzed on an Orbitrap Exploris 240 (Thermo Fisher Scientific) coupled to a Vanquish Neo UHPLC system (Thermo Fisher Scientific). Briefly, 400 ng of peptides were loaded into a PepMap Neo UHPLC column (150 mm × 75 μm) at 250 nL min^–1^ of phase A (0.1% FA). Analytical separation was performed using a gradient of 2–45% of phase B (80% acetonitrile in 0.1% FA) in 30 min. The instrument was operated in data-independent acquisition (DIA) mode, acquiring precursor ions over the *m*/*z* range of 525–825 at a resolution of 60,000, with a maximum injection time of 25 ms and an AGC target of 800%. MS/MS scans were acquired in 50 windows of 6 *m*/*z* with 1 *m*/*z* overlap. Normalized collision energy was set at 28%, and fragment spectra were acquired over the *m*/*z* range of 145–1450 at a resolution of 15,000.

### Proteomics Data Analysis

LC-MS/MS data were analyzed using DIA-NN 2.3.1 with default parameters.^85^ Label-free quantification was performed using the MaxLFQ algorithm through DIAgui.^86^ RPA values were calculated by normalizing each protein’s raw intensity to the total protein intensity of the corresponding group. UpSet plots were generated using the Intervene web tool to visualize shared proteins among NP groups.^87^ PCA analysis was performed in *R*, and heat maps were generated using the ClustVis web tool.^88^ For both analyses, proteins were retained if detected in at least 50% of replicates in any given group. Intensities were then log_2_-transformed, median-centered across samples, and missing values were imputed. Volcano plots were generated in *R* and plotted in OriginPro to visualize differences in protein RPA between conditions. For this analysis, proteins were filtered (>50% detection criterion and RPA > 0.1%), log_2_-transformed, and imputed. To account for the compositional nature of the corona, data were normalized using the centered log-ratio (CLR) transformation. Differential expression was modeled on CLR-transformed values, and p-values were adjusted using the Benjamini–Hochberg false discovery rate (FDR) method. Proteins with FDR < 0.05 and |log_2_FC| ≥ 1 were considered significantly altered.

### Chandler Loop Model

The model was constructed using polyvinyl chloride (PVC) tubes precoated with human serum albumin to create an inert surface (see the Supporting Information for details). The tubes measured 30 cm in length with a 3 mm internal diameter, and they were sealed into a loop using a small PVC segment (4 mm internal diameter) as a sheath. Each loop was filled with 2 mL of fresh whole blood anticoagulated with lithium heparin, leaving approximately 20% of the tube volume unfilled to ensure continuous blood movement during rotation. Blood samples contained either NPs (40 μg mL^–1^), an equivalent volume of PBS as a circulation control, or LPS (100 ng mL^–1^) as a positive control for assays involving immune cell activation. The loops were assembled on a vertical wheel and rotated at 24 rpm for 2 h in a 37 °C water bath to simulate arterial flow conditions. After circulation, 10 mM EDTA was added to halt reactions. An aliquot was immediately analyzed for CD11b expression on leukocytes, while the remaining blood was centrifuged to obtain plasma, which was stored at −80 °C for subsequent ELISA quantification of TAT complex, PF4, C3a, C5a, and PMN elastase levels. Blood that was not subjected to rotation in the Chandler loop experiment served as a baseline for each activation marker. Six individual donors were included for each test condition.

### Hemolysis

After circulation in the Chandler loop system, aliquots of whole blood were collected and diluted 1:50 in PBS. Whole blood without NPs and not subjected to circulation served as the negative control, while positive controls contained 1% Triton X-100 to induce complete hemolysis. The samples were then centrifuged at 1500 × g for 5 min, and the supernatants were collected. Hemoglobin release was quantified using a microplate reader by measuring absorbance at 540 nm. Percent hemolysis was calculated as %:

$$\%\;\mathrm{hemolysis}=100\times \left[\left({\mathrm{OD}}_{\mathrm{sample}}-{\mathrm{OD}}_{\mathrm{neg}}\right)/{\mathrm{OD}}_{\mathrm{pos}}-{\mathrm{OD}}_{\mathrm{neg}}\right)]$$

### Platelet Aggregation

Platelet aggregation in PRP samples was assessed by light transmission aggregometry using a Chrono-Log 490 aggregometer (Chrono-Log Corporation, USA). Platelet counts were adjusted to ∼2.5 × 10^8^ platelets/mL using HEPES-Tyrode buffer. NPs (40 μg mL^–1^) were then incubated with PRP for 30 min at 37 °C, followed by the addition of arachidonic acid (0.5 mM) as an aggregation agonist. Platelet aggregation was expressed as the percent change in light transmittance, where 0% corresponded to PRP and 100% to PPP.

### Contact Activation in PPP

Citrated PPP samples were diluted to 30% (v/v) in HEPES buffer and incubated with NPs (40 μg mL^–1^) for 1 h at 37 °C in a 96-well plate. Dextran sulfate was included as a positive control. Chromogenic substrate S-2302 (300 μM) was then added, and substrate cleavage was monitored at 405 nm using a SpectraMax plate reader (Molecular Devices, USA).

### PT and aPTT

For aPTT measurements, citrated PPP was incubated with or without NPs (40 μg mL^–1^) for 30 min at 37 °C. Clotting was initiated by sequential addition of Actin FS reagent and CaCl_2_ (10 mM), using a plasma:Actin FS:CaCl_2_ volume ratio of 1:1:1. For PT measurements, clotting was triggered by adding Thromborel S reagent to the NP-treated plasma using a reagent:plasma volume ratio of 2:1. Time to clot formation was recorded using a BFT II semiautomated coagulometer (Siemens Healthineers, Germany).

### NP Uptake by Blood Leukocytes

Fluorescently labeled NPs (40 μg mL^–1^) were incubated with 200 μL of whole blood (anticoagulated with 1.5 IU heparin mL^–1^) in a 96-well plate for 2 h at 37 °C. After incubation, erythrocytes were lysed using 1 mL of FACS lysing solution for 15 min, and NP uptake was quantified by flow cytometry using an Accuri C6 flow cytometer (BD Biosciences, USA). Granulocytes and monocytes were gated based on their forward scatter and side scatter profiles, with cell debris and doublets removed using standard procedures. NP internalization was represented as the percentage of NP-positive cells within each gated population.

### CD11b Expression by Flow Cytometry

Following circulation in the Chandler loop system, CD11b expression was assessed by incubating 100 μL of blood with 10 μL of anti-CD11b-PE antibody for 30 min at 37 °C. Erythrocytes were then lysed by adding 1 mL of FACS lysing solution for 15 min. Cells were then centrifuged with 500 × g for 10 min and resuspended in 1 mL of PBS. CD11b expression was subsequently analyzed by flow cytometry, and results were reported as median fluorescence intensities (MFI) derived from the fluorescence-intensity distributions.

### Plasmatic Markers by ELISA

After circulation in the Chandler loop system, plasma levels of the TAT complex, PF4, C3a, C5a, and PMN elastase were measured using commercial ELISA kits following the manufacturer’s instructions. For C3a and C5a, zymosan (40 μg mL^–1^) was incubated with plasma as a positive control prior to ELISA analysis. Additionally, C3a levels were measured in PPP samples incubated with NPs for 1 h at 37 °C. PPP was anticoagulated using argatroban (0.1 mg mL^–1^), a direct thrombin inhibitor that does not interfere with the complement system. After incubation, 10 mM EDTA was added to halt reactions. Absorbance was measured using a SpectraMax plate reader.

### NP Cytotoxicity

NPs (40 μg mL^–1^) were added to 1 × 10^5^ PBMCs in cRPMI in 96-well plates. PBMCs containing staurosporine or PBS served as positive and negative controls, respectively. The mixtures were incubated for 24 h at 37 °C under a 5% CO_2_ atmosphere. After incubation, cells were collected, washed with ice-cold PBS, and resuspended in Annexin V binding buffer. Cells were then stained with Annexin V and propidium iodide at the recommended volumes and incubated for 15 min in the dark. Samples were analyzed by flow cytometry to quantify viable cells, early apoptotic cells, and late apoptotic/dead cells.

### Cytokine Release from PBMCs

NPs (40 μg mL^–1^) were added to 1 × 10^5^ PBMCs in cRPMI in 96-well plates. PBMCs containing LPS (100 ng mL^–1^) or PBS served as positive and negative controls, respectively. The mixtures were incubated for 24 h at 37 °C under a 5% CO_2_ atmosphere. After incubation, samples were centrifuged and supernatants were collected for cytokine analysis. Cytokine secretion was quantified using a five-plex bead-based assay run on the Luminex xMAP platform (Luminex Corporation, USA).

### Leukocyte PCA

NPs (40 μg mL^–1^) were added to 1 × 10^7^ PBMCs in cRPMI in 6-well plates. PBMCs containing LPS (100 ng mL^–1^) or PBS served as positive and negative controls, respectively. The mixtures were incubated for 24 h at 37 °C under a 5% CO_2_ atmosphere. After incubation, cells were washed and resuspended in HEPES buffer containing 6.6 mM CaCl_2_ to support coagulation. Equal volumes of the resuspended cells and PPP were then mixed, and the time to clot formation was measured using a coagulometer.^61,79^

### Leukocyte Proliferation

NPs (40 μg mL^–1^) were added to 1 × 10^5^ PBMCs in cRPMI in 96-well plates in the presence or absence of PHA-M (10 μg/mL). PBMCs without NPs (with or without PHA-M) served as controls. The mixtures were incubated for 72 h at 37 °C under a 5% CO_2_ atmosphere. Lymphocyte proliferation was assessed by MTT assay, measuring absorbance at 570 nm in a plate reader.^61,79^

### Statistics

NP characterization data and measurements obtained from blood, plasma, PBMCs, and platelets were processed in OriginPro and are presented as bar graphs showing the mean ± SD. Potential outliers were identified using Grubbs’ test. Intergroup differences were analyzed using one-way ANOVA with Tukey’s posthoc test when the assumptions of normality (Shapiro–Wilk test) and homogeneity of variances (Levene’s test) were satisfied. When data were normally distributed but variances were unequal, Welch’s ANOVA followed by the Games–Howell posthoc test was applied. When both assumptions were violated, data were evaluated using the nonparametric Kruskal–Wallis test followed by Dunn’s posthoc test for pairwise comparisons. To facilitate visualization, statistical comparisons were displayed only between each experimental group and the relevant control condition in each case. Statistical significance was set at *p* < 0.05, with *p < 0.05, **p < 0.01, and ***p < 0.001.

## Supplementary Material



## Notes

Supplementary methods, description of the Chandler loop system setup; Figure S1, assessment of NP aggregation; Figure S2, assessment of HSA precoating stability; Figure S3, protein corona analysis by volcano plots; Figure S4, validation of the Chandler loop system; Figure S5, assessment of NP cytotoxicity; Figure S6, assessment of NP-induced hemolysis and platelet activation (PDF)
