# Supplementary material for: Albumin Precoating of Nanoparticles: A Reliable Stealth Strategy?
Source: ACS Omega. 2026 Aug 17;11(34):51281–95. doi: 10.1021/acsomega.6c04297 (PMC13625134; doi:10.1021/acsomega.6c04297)
Supplement: Supplementary file 1 [file ao6c04297_si_001.pdf]

Supplementary Information

Albumin Precoating of Nanoparticles: A Reliable Stealth Strategy?

Vinicius S. Guido,[1] André F. Lima,[1] Natasha Mina,[1] Jackelinne Y. Hayashi,[1] Ismael F. Lima,[2] Leo K. Iwai,[2] Maria Luiza V. Oliva,[1] Fernando L. A. Fonseca,[3,4] Alexandre K. Tashima,[1,5] Alioscka A. Sousa[1,*]

[1] Department of Biochemistry, Federal University of São Paulo (UNIFESP), São Paulo, SP 04044-020, Brazil

[2] Laboratory of Applied Toxinology, Center of Toxins, Immune-response and Cell Signaling LETA/CeTICS, Butantan Institute, São Paulo, SP 05503-900, Brazil

[3] Pharmaceutical Sciences Department, Federal University of São Paulo (UNIFESP), Diadema, SP 09913-030, Brazil.

[4] Clinical Laboratory Department, Faculty of Medicine of ABC (FMABC), Santo Andre, SP 09060-650, Brazil

[5] Laboratory of Mass Spectrometry Applied to Biomolecules, Federal University of São Paulo (UNIFESP), São Paulo, SP 04039-032, Brazil

*Corresponding author: alioscka.sousa@unifesp.br

Chandler loop system

The Chandler loop model was constructed using polyvinyl chloride (PVC) tubes pre-coated with human serum albumin to create an inert surface. The tubes measured 30 cm in length with a 3 mm internal diameter, and they were sealed into a loop using a small PVC segment (4 mm internal diameter) as a sheath. Each loop was filled with 2 mL of fresh whole blood anticoagulated with lithium heparin, leaving approximately 10% of the tube volume unfilled to ensure continuous blood movement during rotation.

When exposed to whole blood, we found that uncoated PVC tubes significantly increased activation markers for platelets, leukocytes, and the coagulation and complement systems compared to baseline controls (**Suppl. Fig. S4**). This indicated that uncoated PVC tubing was unsuitable for Chandler loop assays. To improve hemocompatibility, previous studies have employed heparin coatings, which enhance antithrombin activity, or synthetic MPC polymers, which mimic phospholipid polar groups to create an antifouling surface.[1, 2] As a more convenient and cost-effective alternative, we coated the tubes with purified albumin to provide a similarly inert surface. Albumin coating was achieved by loading the tubes with 1 mL of a 10 mg/mL albumin solution and rotating overnight, followed by washing with Milli-Q water to remove unbound protein. This approach resulted in surfaces that met hemocompatibility criteria across all tested parameters, showing virtually no differences compared to the baseline control (**Suppl. Fig. S4**). Moreover, the coated tubes responded appropriately to LPS exposure, with significant increases in activation marker levels relative to baseline. The only marker that did not increase was TAT, which aligns with the fact that LPS is not a strong direct activator of the plasma coagulation cascade. Although we did not perform a direct comparison with heparin- or MPC polymer-coated surfaces, our findings suggest that the albumin coating provided comparable performance under the tested experimental conditions.

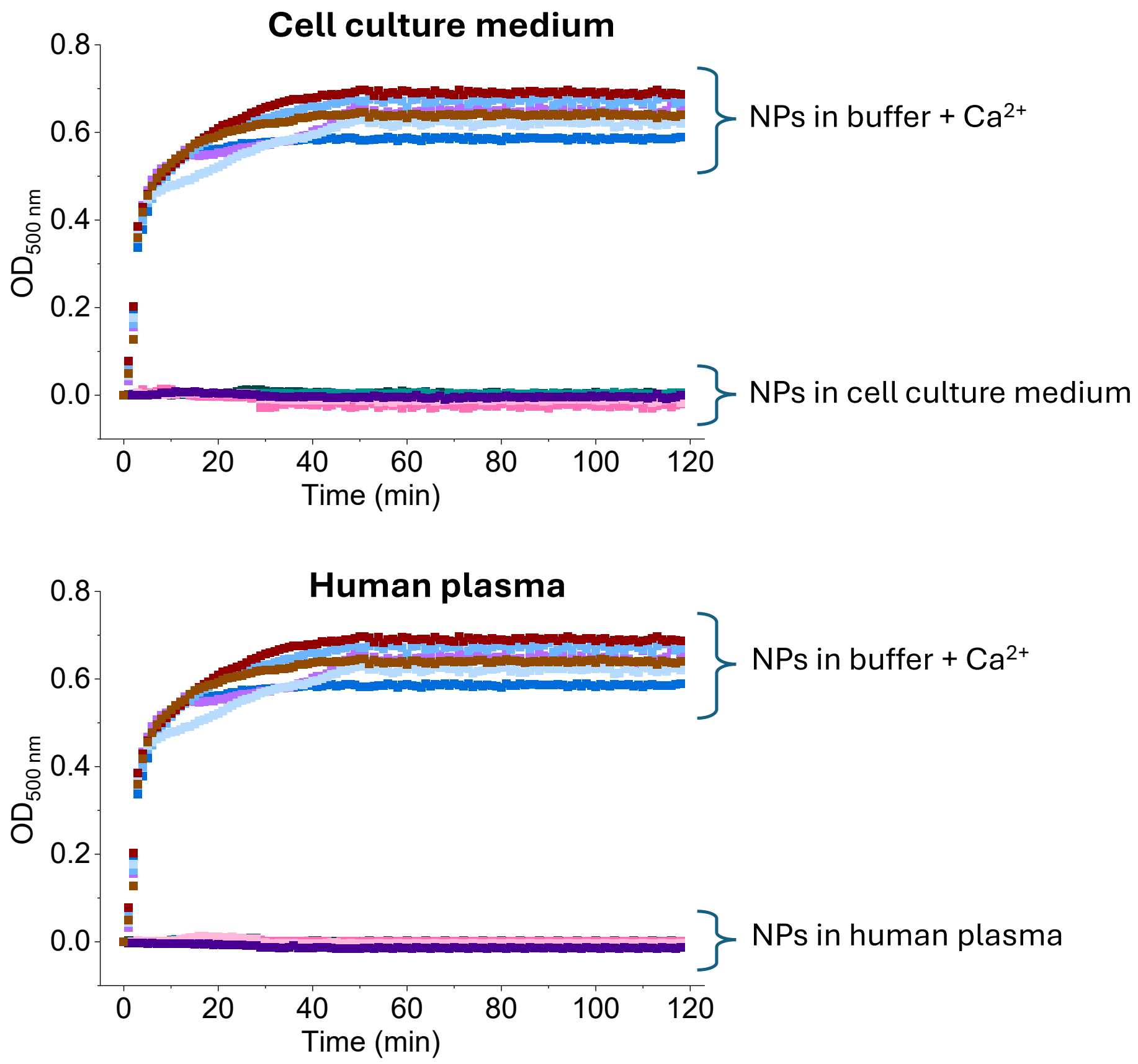

Suppl. Figure S1. NP aggregation evaluated by optical turbidimetry. Uncoated (C-Uncoated, S-Uncoated) and HSA-coated (C-HSA-f, C-HSA-df, S-HSA-f, S-HSA-df) nanoparticles (40 µg/mL) were separately dispersed in cell culture medium or 30% human plasma, and aggregation was monitored by turbidimetry (500 nm) for 24 h. NPs dispersed in buffer supplemented with a high concentration of Ca^{2+} (25 mM) served as a positive control for aggregation. The results showed that NPs aggregated in the presence of Ca^{2+}, whereas no aggregation was observed in either cell culture medium or human plasma. Data are shown for the first 2 h for clarity; the same trend was maintained throughout the 24 h test period.

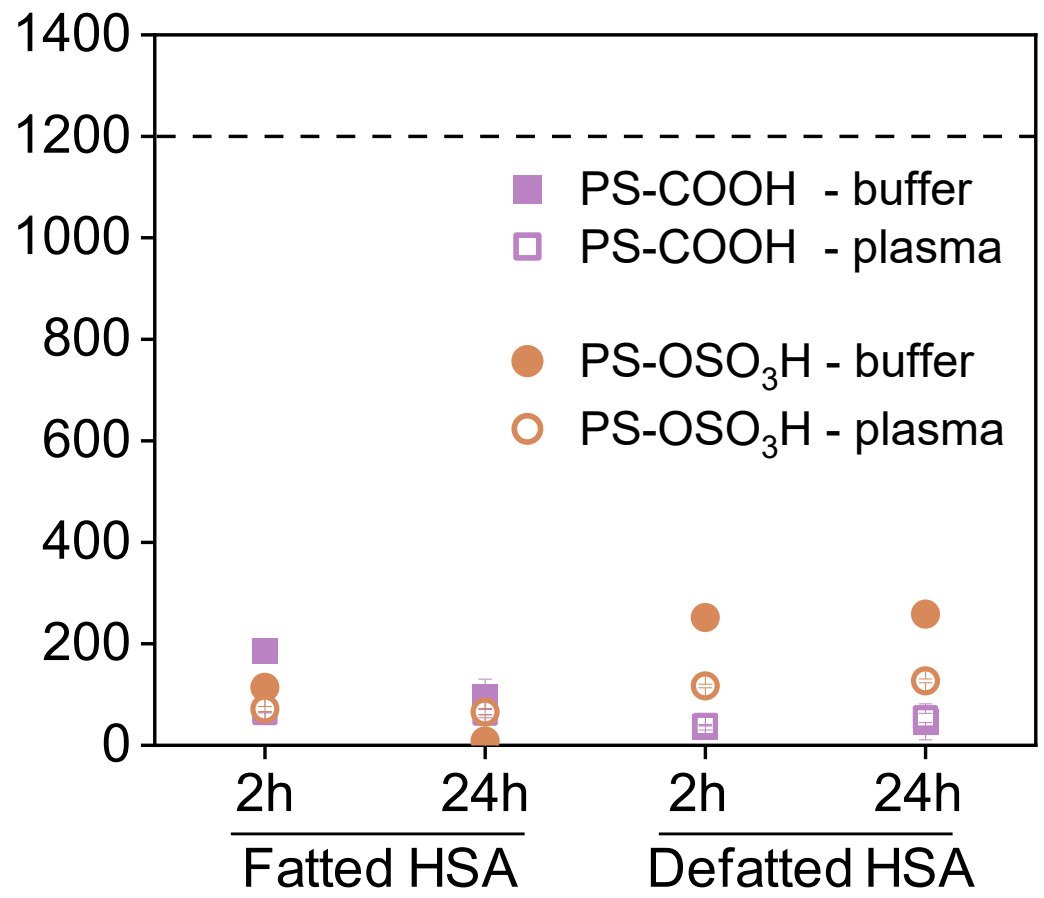

Suppl. Figure S2. Evaluation of HSA coating stability in human plasma. Fatted and defatted HSA were labeled with Atto 488 and used to prepare HSA-coated NPs. These particles were then incubated in either buffer or 50% human plasma for 2 or 24 h. Following centrifugation, fluorescence in the supernatant was measured to assess potential detachment of HSA. The dashed line indicates the approximate fluorescence expected if all bound HSA were to be detached from the NP surface. In all cases, a small amount of fluorescence in the supernatant of plasma samples was observed, which was equal to or lower than that observed in the control buffer. Moreover, fluorescence levels remained constant between 2 and 24 h. Taken together, these results suggest that the low fluorescence detected in the presence of plasma reflects some NP loss during centrifugation rather than HSA exchange with plasma proteins. Therefore, it can be concluded that the HSA precoating was largely stable and remained bound even in biological fluid. However, because this method provides only an approximate estimate, a minor exchange of HSA with plasma proteins cannot be excluded.

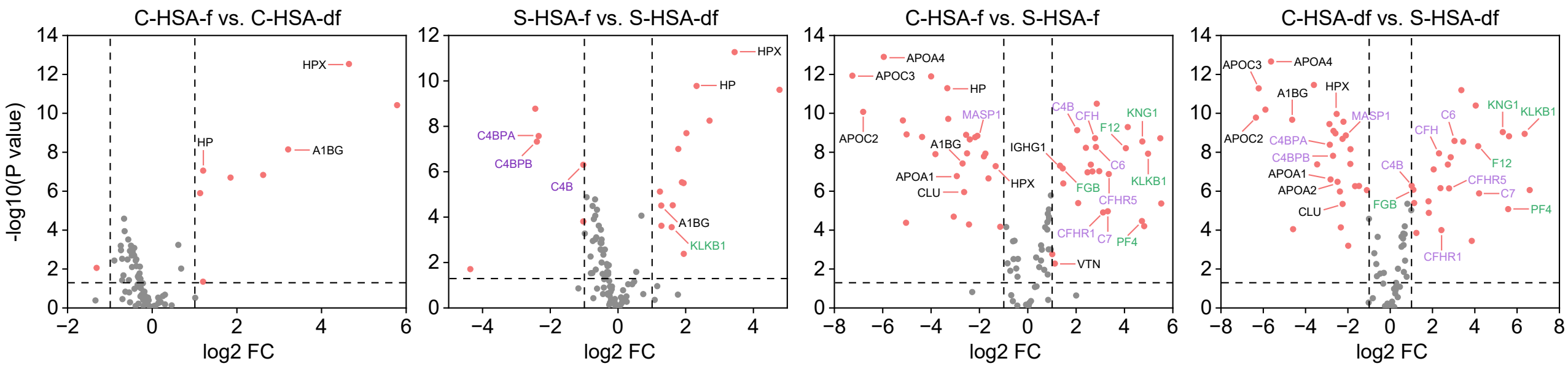

Suppl. Figure S3. Volcano plots showing statistically significant changes in protein RPAs between HSA-precoated NP formulations. Differentially represented proteins are highlighted, focusing on the top 10 most abundant corona proteins (black labels), complement proteins (purple labels), and coagulation factors (green labels). While enrichment or depletion of several complement components was observed, core elements of the classical and alternative pathways, including C1q, C3, and factor B, were not detected. MASP1, a central component of the lectin pathway, was enriched in some coronas; however, its relative abundance was low (RPA ~0.1%). The analysis also revealed enrichment of contact activation factors of the intrinsic coagulation pathway, including factor XII (F12), plasma kallikrein (KLKB1), and high-molecular-weight kininogen (KNG1), in the coronas of C-HSA-f and C-HSA-df.

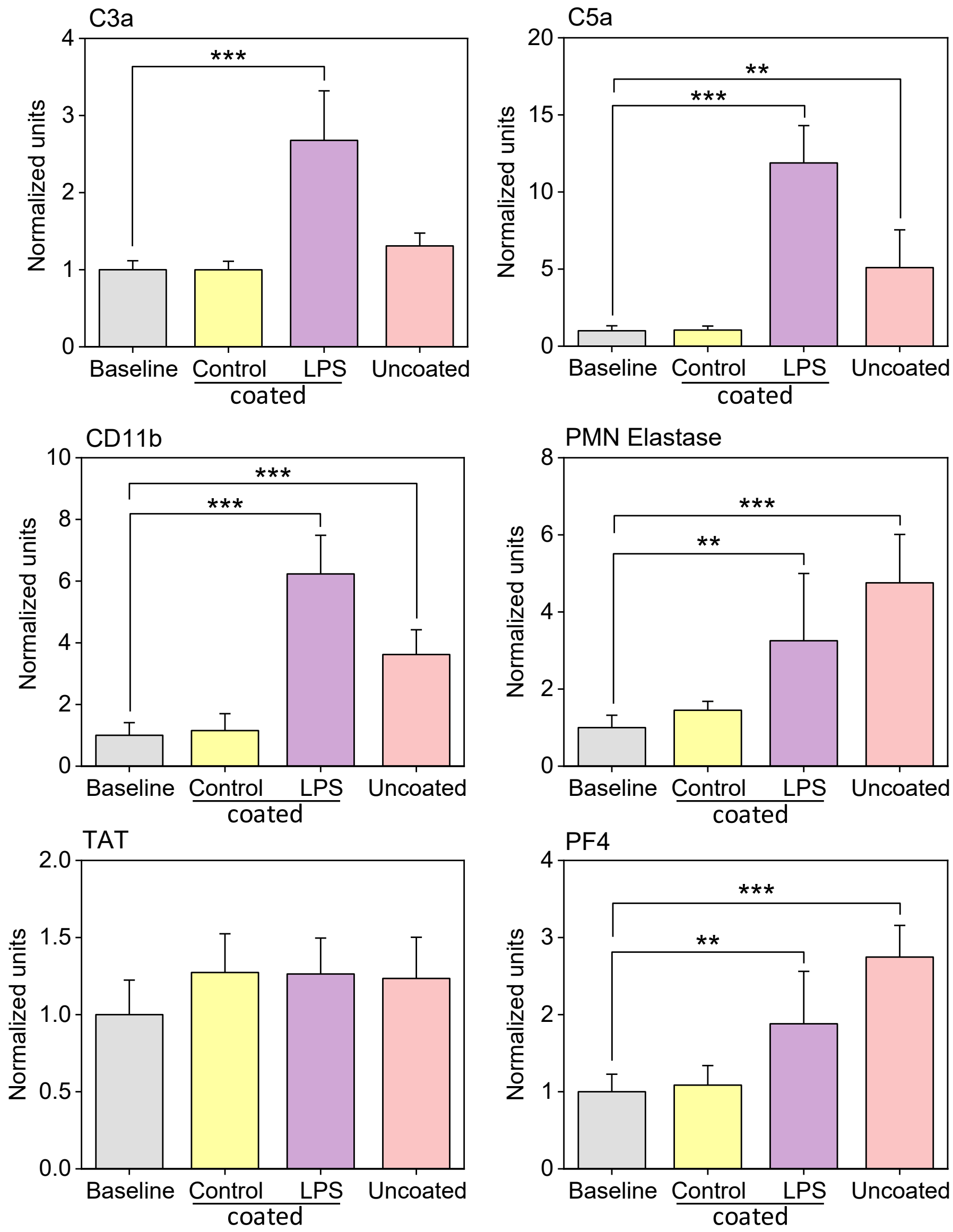

Suppl. Figure S4. Validation of the Chandler loop setup. Whole blood (1.5 IU heparin mL^{-1}) was loaded into either HSA-coated or uncoated PVC tubing and rotated for 2 h at 37°C. As a positive control, blood in HSA-coated tubes was supplemented with LPS (100 ng mL^{-1}). After circulation, plasma was analyzed for C3a, C5a, PMN elastase, TAT, and PF4 by ELISA, and CD11b expression on leukocytes was assessed by flow cytometry. Blood that was not subjected to rotation served as the baseline for each activation marker. Six individual donors were used for each test condition, except for uncoated tubing, where four donors were used.

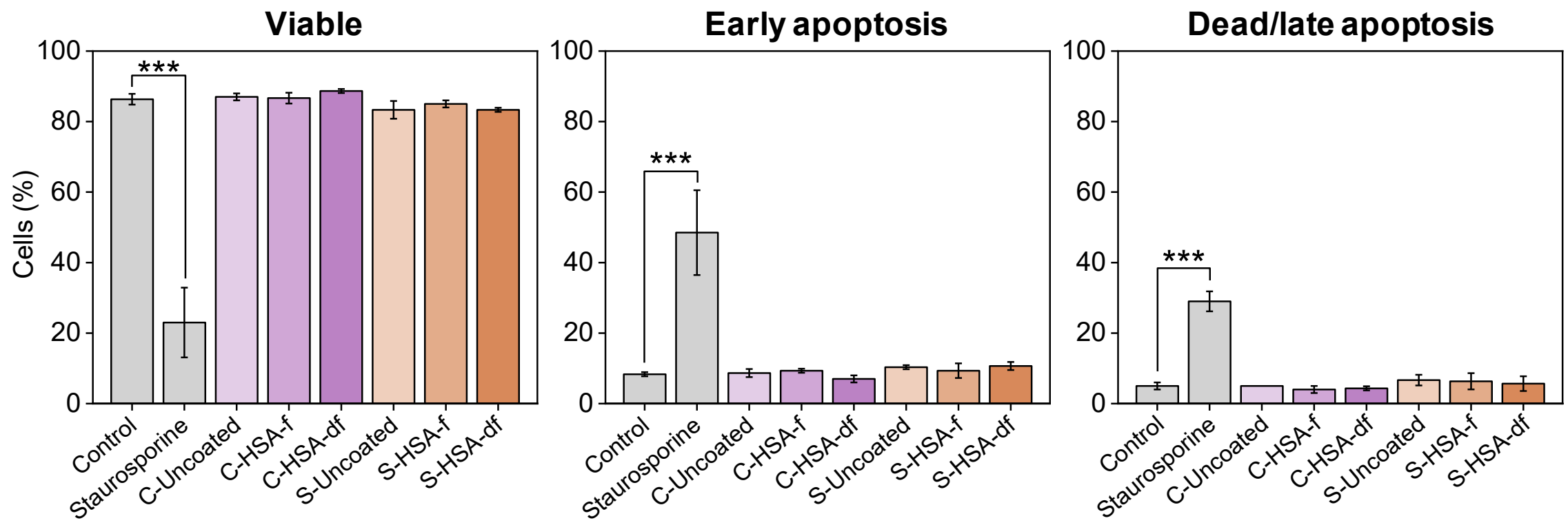

Suppl. Figure S5. NP-induced cytotoxicity. PBMCs were incubated with NPs for 24 h at 37°C, and cytotoxicity was assessed by Annexin V/PI staining followed by flow cytometry. Buffer without NPs served as the negative control and staurosporine (100 ng mL^{-1}) as the positive control.

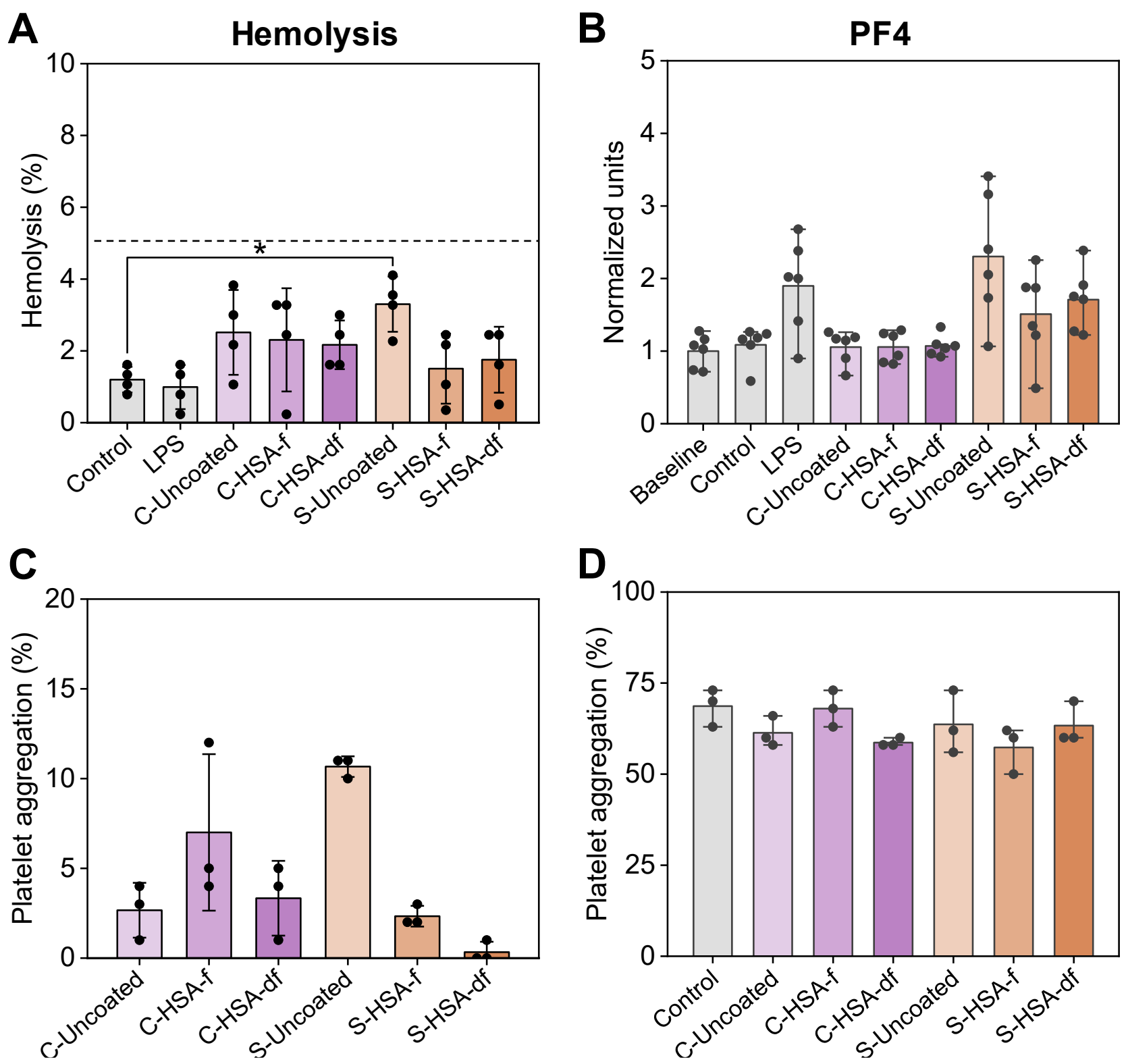

Suppl. Figure S6. NP-induced hemolysis and platelet activation. (A) Hemolysis assessed in whole blood. NPs were circulated with whole blood in the Chandler loop system, and hemoglobin release was quantified by measuring absorbance at 540 nm. Control and LPS refer to blood without NPs and blood treated with LPS (100 ng mL^{-1}), respectively. The dashed line marks the 5% threshold, above which the tested material

is considered hemolytic. Statistical analysis was performed using one-way ANOVA followed by Tukey's post hoc test. (B) Platelet activation in whole blood. NPs were circulated with whole blood in the Chandler loop system, followed by quantification of PF4 release in the plasma fraction using ELISA. Baseline and Control refer to blood without NPs before and after circulation, respectively. Blood treated with LPS (100 ng mL^{-1}) was included for comparison. Results were normalized to the baseline. Statistical analysis was performed using Welch's ANOVA followed by the Games–Howell post hoc test, which revealed no significant differences. (C,D) Platelet activation in PRP. PRP was incubated with or without NPs for 0.5 h at 37°C, followed by the addition of arachidonic acid and assessment of platelet aggregation by light transmission aggregometry. Results in (C) show that none of the NPs triggered aggregation independently, except for S-Uncoated, which showed a tendency toward platelet activation. Results in (D) show that none of the NPs interfered with arachidonic acid–induced aggregation.

References

(1) Ekdahl, K. N.; Fromell, K.; Mohlin, C.; Teramura, Y.; Nilsson, B. A Human Whole-Blood Model to Study the Activation of Innate Immunity System Triggered by Nanoparticles as a Demonstrator for Toxicity. *Sci. Tech. Adv. Mat.* **2019**, *20*, 688-698.

(2) Asif, S.; Asawa, K.; Inoue, Y.; Ishihara, K.; Lindell, B.; Holmgren, R.; Nilsson, B.; Rydén, A.; Jensen-Waern, M.; Teramura, Y. Validation of an Mpc Polymer Coating to Attenuate Surface-Induced Crosstalk between the Complement and Coagulation Systems in Whole Blood in in Vitro and in Vivo Models. *Macrom. Biosc.* **2019**, *19*, 1800485.
